# Mitochondrial plastid DNA can cause DNA barcoding paradox in plants

**DOI:** 10.1038/s41598-020-63233-y

**Published:** 2020-04-09

**Authors:** Hyun-Seung Park, Murukarthick Jayakodi, Sae Hyun Lee, Jae-Hyeon Jeon, Hyun-Oh Lee, Jee Young Park, Byeong Cheol Moon, Chang-Kug Kim, Rod A. Wing, Steven G. Newmaster, Ji Yeon Kim, Tae-Jin Yang

**Affiliations:** 1 Department of Plant Science, Plant Genomics and Breeding Institute, and Research Institute of Agriculture and Life Sciences, College of Agriculture and Life Sciences, Seoul National University, Seoul, 08826 Republic of Korea; 2 Phyzen Genomics Institute, Seongnam, 13558 Korea; 3 Herbal Medicine Research Division, Korea Institute of Oriental Medicine, 1672 Yuseong-daero, Yuseong-gu, Daejeon, 34054 Republic of Korea; 4 Genomics Division, National Institute of Agricultural Sciences, Jeonju, 54874 Republic of Korea; 5 Arizona Genomics Institute, School of Plant Sciences, The University of Arizona, Tucson, AZ USA; 6 NHP Research Alliance, College of Biological Sciences, University of Guelph, Guelph, Ontario Canada; 7 Department of Food Science and Technology, Seoul National University of Science and Technology, Seoul, 01811 Korea

**Keywords:** Mitochondrial genome, Genome evolution, Plant biotechnology, Plant evolution, Plant genetics

## Abstract

The transfer of ancestral plastid genomes into mitochondrial genomes to generate mitochondrial plastid DNA (MTPT) is known to occur in plants, but its impacts on mitochondrial genome complexity and the potential for causing a false-positive DNA barcoding paradox have been underestimated. Here, we assembled the organelle genomes of *Cynanchum wilfordii* and *C. auriculatum*, which are indigenous medicinal herbs in Korea and China, respectively. In both species, it is estimated that 35% of the ancestral plastid genomes were transferred to mitochondrial genomes over the past 10 million years and remain conserved in these genomes. Some plastid barcoding markers co-amplified the conserved MTPTs and caused a barcoding paradox, resulting in mis-authentication of botanical ingredients and/or taxonomic mis-positioning. We identified dynamic and lineage-specific MTPTs that have contributed to mitochondrial genome complexity and might cause a putative barcoding paradox across 81 plant species. We suggest that a DNA barcoding guidelines should be developed involving the use of multiple markers to help regulate economically motivated adulteration.

## Introduction

Although plastids are well conserved in most plants, their genomes can potentially confound barcoding analysis and cause species mis-identification. Plastid genomes range from those with moderate intraspecific diversity that are maternally inherited^1^^–^^5^ to more diverse plastid genomes in plants with biparentally inherited heteroplasmy^6^. Additional genomic diversity occurs through a mechanism involving horizontal plastid genome transfer into the mitochondrial and nuclear genomes, giving rise to mitochondrial sequences of plastid origin (MTPTs) and nuclear genome sequences of plastid origin (NUPTs), respectively^7–11^. Such complexity in the genome can cause mis-interpretation of sequences and subsequent mis-identification of species. For example, these is a higher probability of DNA barcoding misidentification in animal species with promiscuous DNA in which there is co-amplification of nuclear mitochondrial *COI* pseudogenes^8^. The occurrence of this genomic mechanism in plants is not well documented and presents a clear gap in our application of DNA barcoding in plants.

There are key uncertainties concerning our knowledge of the occurrence of plastid-mitochondrial genome flux and how it may influence the interpretation of DNA barcodes. Although more than 300 nuclear genomes and thousands of chloroplast genomes from plants have been documented, only 79 mitochondrial genomes have been sequenced in plants (GenBank, Dec. 2017). By contrast, more than 8,000 animal mitochondrial genomes have been reported and are utilized for evolutionary and taxonomic studies^12^. Compared to other organisms, plant mitochondrial genomes exhibit large variations in genome size and have complex structures. They can also contain repeat-mediated multi-chromosomes, even in a single cell, making the genome difficult to assemble and unsuitable for use as a barcoding target^13^. The complexity of the mitochondrial genome may lead to considerable plastid–mitochondrial genome flux in plants^14^.

DNA barcoding may not be fit-for-purpose if there are misidentifications of commercial species in quality control systems. Identity testing methods must be able to accurately detect target ingredients to verify claims on labels. Accidental contamination and economically motivated adulteration (EMA) of herbal products have become common, serious threats to the herbal industries^15,16^. Such fraud costs the industry approximately $10–$15 billion globally every year due to the need to authenticate products and eliminate EMA targets^15^. More broadly, such fraud also damages the market for natural products by eroding consumer trust. DNA barcoding based on plastid (or chloroplast) and 45S nuclear ribosomal DNA (45 S rDNA) has been suggested as a credible, appropriate means to identify plant species ingredients in food and natural health products^17–19^. However, the misapplication of such DNA markers can be highly problematic for the industry. In February 2015, the New York State Attorney General’s Office accused major herbal product retailers of selling adulterated products based on DNA barcode data and prohibited the retailers from selling the products^20^. However, the products were restored to market after an investigation determined that they had been produced in compliance with the guidelines of the US Food and Drug Administration and that barcode testing was not a scientifically validated test for regulatory or commercial identity testing applications. There is a considerable likelihood for both false positive and negative tests, as no guidelines have been published that address criteria such as specificity, sensitivity, limit of detection, applicability, repeatability and reproducibility^21^.

The commercial use of a scientific test method before it is validated can cause serious economic damage to specific brands, businesses and entire industries. For example, in April 2015, there was a false-positive ingredient identity test recorded in a Korean health functional food that has a label claim for an extract of the medicinal plant *Cynanchum wilfordii* (Cw), which is used to treat menopausal disorders^22^. Cw is an widely distributed herbal plant in Korea that is registered and approved as an ingredient for health functional foods by the Ministry of Food and Drug Safety in Korea^23^. Cw is reported to have several physiological effects such as enhancing immunity, inhibiting benign prostatic hyperplasia and anti-osteoporosis effects in animal models^24–26^. Cw has also been investigated as a functional food for reducing fat accumulation and liver damage^27^. An ethanol extract of Cw increased HDL-cholesterol levels and reduced the atherogenic index in rats and mice that were fed a high fat and high cholesterol diet^28,29^. Very recently, Youn *et al*. reported that an ethanol extract of Cw significantly reduced total cholesterol, apolipoprotein B and cholesteryl ester transfer protein levels in mild hypercholesterolemia subjects^30^. *Cynanchum auriculatum* (Ca) is a closely related plant species that is utilized as traditional Chinese medicine in China^31^. Ca has anti-tumor activity^32^ with both gastroprotective^33^ and antioxidant effects^34^. Ironically, Ca is not a desirable ingredient because it has not yet been registered as a food material in Korea. On the other hand, Ca has been used for local food and folk medicine in China^35,36^. Cw extract was a best-selling health functional food in Korea in 2012–2015. However, sales of the most popular Cw product plummeted after accusations of contamination with Ca instead of Cw, although both have similar functions and metabolite profiles^33,36^. At that time, two plastid DNA markers including intergenic targets between *trnH* and *psb*A genes as well as intron region of *trnL* were reported for discrimination of Cw and Ca^37,38^. The manufacturer of this product was sued and suffered public defamation for product adulteration by issuing from the Korea Consumer Agency who used one of the two DNA barcoding markers to make an adulteration claim. This adulteration claim was refuted, and the case was acquitted by the Korean Supreme Court due to the lack of properly validated scientific methods and the use of only one or two plastid DNA markers, which were inadequate for identifying these ingredients, as these barcodes could not discriminate these two closely related plant species. However, the industrial use of Cw products has sharply declined and its production has not yet recovered. Moreover, many innocent Cw farmers are still under suffering from an obligate regulation involving the use of a single real-time PCR marker, *matK*, with some false-positive detection defects derived from the plastid–mitochondrial genome flux in these taxa^39^.

The goal of the current study was to investigate plastid–mitochondrial genome flux and its impact on the herbal medicinal industry using ingredients from *Cynanchum* species as a case study. More specifically, we assembled the complete plastid and mitochondrial genomes of two *Cynanchum* species in order to assess the occurrence of plastid–mitochondrial genome flux in these species. In addition, we analyzed 81 mitochondrial genomes to explore lineage-specific MTPT patterns, which may contribute to mitochondrial genome diversity, as well as MTPTs capable of causing mis-authentications while employing DNA barcoding. Finally, we propose a set of recommended principals for developing guidelines for eliminating EMA using a multiple marker system.

## Results

### Plastid and mitochondrial genome sequences of ***C. wilfordii*** and ***C. auriculatum***

**Figure 1:**
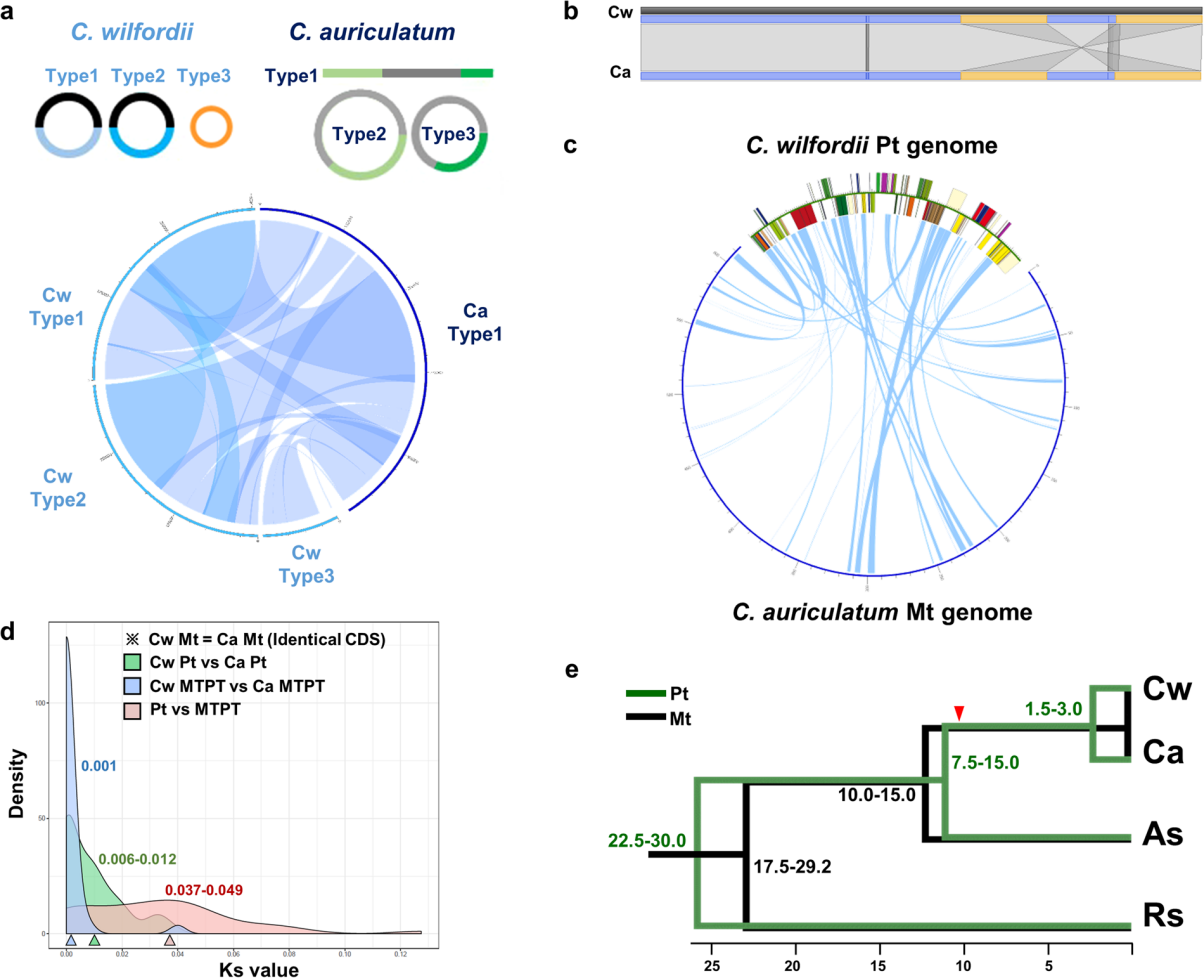
Plastid–mitochondrial genome structure, flux and evolution in *Cynanchum* species. (**a)** Schematic representation of each type of mitochondrial genome and comparison in *C. wilfordii* (Cw) and *C. auriculatum* (Ca). Each line represents a type of mitochondrial genome (of the three per species), and genomic blocks with homology are connected at both the intra- and interspecies levels. The Circos plot shows synteny between the mitochondrial genomes of Cw and Ca. **(b)** Comparison of the overall plastid genome structures of Cw and Ca. Yellow rectangles indicate inverted repeat blocks, and small lines indicate repeats between these blocks. **(c)** Circos plot comparing the plastid and mitochondrial genomes of the two *Cynanchum* species. **(d)** Density plot of the rates of nucleotide substitution between homologous genes (Ks values) in the plastid and mitochondrial genomes of the two *Cynanchum* species. The green line shows Ks values between the plastid genes of Cw and Ca, and the gray line shows those between the MTPTs of the two species, whereas the red line shows Ks values between the MTPT and its plastid counterpart for each species. The mode value of Ks is marked with a triangle in each case (Supplementary Table 3). **(e)** Estimation of divergence time among four Apocynaceae species based on Ks values of plastid (green) and mitochondrial genes (black). The red triangle indicates the estimated time of MTPT insertion in the common ancestor of the two *Cynanchum* species. The synonymous substitution rates per year per base are 2 × 10^−9^ for plastid and 0.6 × 10^−9^ for mitochondria. As, *Asclepias syriaca*; Rs, *Rhazya stricta*.

**Figure 2:**
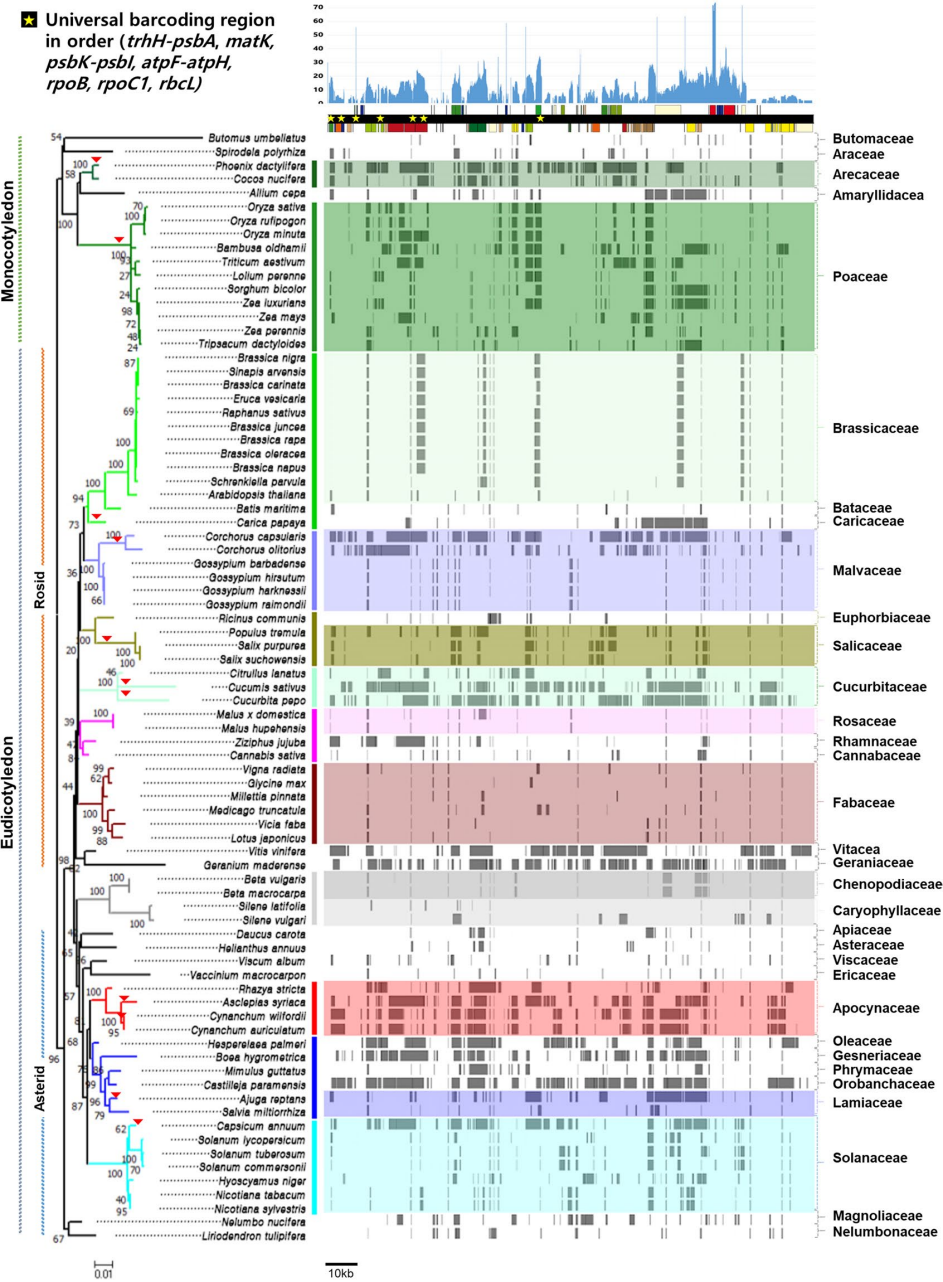
Schematic representation of MTPTs in 81 plant mitochondrial genomes. The *Arabidopsis thaliana* plastid genome sequence was used as a backbone for the MTPTs in the mitochondrial genome of each species. The depths of MTPTs, indicating the frequency of *Arabidopsis* nucleotides among the 81 species, are represented on the linear genome map of the *Arabidopsis* plastid. Seven universal land plant barcoding regions^14^ are marked on the map with yellow stars following their location and order in the genome. The MTPT fragments in each mitochondrial genome are represented as gray blocks. tRNA and rRNA regions are not represented. The phylogenetic relationship was reconstructed using the *matR* sequences of 81 mitochondrial genomes. Areas of recent putative plastid–mitochondrial genome flux are marked with red triangles on the tree.

We assembled the complete plastid^40,41^ and mitochondrial genomes of Cw and Ca using low-coverage whole-genome sequencing (WGS) (Table 1, Supplementary Fig. 1). For Cw, we obtained three type of circular forms of the mitochondrial genome. Type 1 and 2 shared 50% conserved downstream sequence, while type 3 shared no sequence homology with the other two. The total genome lengths were 379,601 bp, 352,774 bp and 111,332 bp for types 1, 2 and 3, respectively. The Ca mitochondrial genome was assembled into one linear major chromosome 652,279 bp in length, along with two minor types of circular forms derived from different recombination events of the three compartments. Both mitochondrial genomes showed large-scale collinearity with some structural rearrangement (Fig. 1a). The collinear regions showed high sequence similarity, and the sequences of all the mitochondrial genes were identical except for the copy number of the *atp9* gene, which was two and one in Cw and Ca, respectively. The plastid genomes of both species showed overall collinearity, with 97.5% similarity (Fig. 2b).

**Table 1:** NGS and organelle genome information for Cw and Ca.

	Cw	Ca
**NGS information**		
Total amount of raw NGS data (bp)	1,122,301,531	1,165,960,744
Total number of raw reads	3,737,112	3,883,572
Total amount of trimmed NGS data (bp)	724,363,954 (64.54%)	801,590,026 (86.28%)
Total number of trimmed reads	3,064,122 (81.99%)	3,350,936 (68.75%)
Plastid genome (bp)	161,241	160,840
GC contents	37.77%	37.76%
Number of annotated genes	114	114
**Mitochondrial genome (bp)**		
Type 1	379,601	652,279
Type 2	352,774	531,558
Type 3	111,332	426,556
GC contents	43.72%	43.78%
**Number of annotated genes**		
Type 1	96	128
Type 2	84	
Type 3	34

### MTPTs in the two *Cynanchum* species

In the plastid genomes, 34.3% and 37.7% of Cw and Ca sequences, respectively, showed homology with mitochondrial genome sequences of the same species (Fig. 1b). Almost 50% of plastid protein-coding genes—36 of the 75 genes—were identified in MTPTs of both *Cynanchum* species (Supplementary Table 2). Two universal DNA barcoding genes from the plastid genome, *matK* and *rpoB*, were also identified in MTPTs of both species: while *rpoB* was identified in the mitochondrial genomes in truncated forms, 135 bp and 297 bp, the complete genic region of *matK* was found in the mitochondrial genomes. Although the MTPT and its plastid counterparts showed relatively high diversity of insertions and deletions (InDels), the sequence similarity between homologous sequences in the two *Cynanchum* species was high (94.5%). The *matK* genes in the plastids of the two species shared 99.2% sequence similarity, and those in the MTPTs shared 99.6% sequence similarity (Supplementary Fig. 2a and Table 3).

### Nucleotide substitution rates in the plastid and mitochondrial genomes

We calculated the nucleotide substitution rates of homologous genes (synonymous substitutions per synonymous site, Ks) between species in the plastid, mitochondrion and MTPT regions (Fig. 1c and Supplementary Table 4). When we performed interspecies comparisons of each organelle genomic content between the two *Cynanchum* species, the mode of the Ks values of the 75 plastid genes was approximately 0.006–0.012 and that of the MTPT regions was ~0.001. The mitochondrial genes of both species were identical, and thus the Ks values were 0. However, when we performed intraspecies comparisons of plastid genes and their MTPT counterparts, the Ks values were much higher and broadly distributed (0.000–0.127) in both species.

We calculated the divergence times of the two *Cynanchum* species and members of two related genera, *Asclepias syriaca* and *Rhazya stricta*, based on the Ks values (Fig. 1d) of their plastid and mitochondrial genes. The divergence rate calculated from the mitochondrial genome was much slower than that calculated from the plastid genome. *R. stricta* first diverged ~17.5–30.0 million years ago (MYA), and *A. syriaca* and the two *Cynanchum* species separated next at ~7.5–15.0 MYA. The two *Cynanchum* species were estimated to have diverged ~1.5–3.0 MYA based on plastid genome sequence divergence, although their mitochondrial genes are identical. The MTPT sequence showed 90% sequence similarity between *A. syriaca* and *Cynanchum* species and 99% sequence similarity between Cw and Ca. We found that a recent episode of elevated plastid–mitochondrial genome flux occurred ~10.7 MYA in the common ancestor of Cw and Ca based on Ks values between plastid and MTPT counterparts.

### Plastid genome flux into the mitochondrial genomes of angiosperms

We investigated MTPTs in the mitochondrial genome sequences of 81 flowering plants (Supplementary Table 4). All 78 protein-coding genes in the plastid genome of *Arabidopsis thaliana* were identified at least once as MTPTs among the 81 plant mitochondrial genomes examined (Fig. 2). *rbcL* was the most frequently detected MTPT, followed by *atpB*, *psaA*, *psaB*, *psbC*, *psbD*, *rpl2*, *rpl23*, *rpoB*, *rps7*, *rps12* and *ycf2*, which were each identified in more than 20 plant species. The international recommendations for barcoding candidate regions for land plants include seven plastid targets, four genic and three intergenic regions^42^ Among the four genic regions, *rbcL* and *rpoB* belonged to the most frequent MTPT group; *rpoC1* was grouped in the moderately frequent group, as it was found in more than 10 species; and *matK* was rarely detected as an MTPT. The three intergenic regions (*atpF*-*atpH*, *psbK*-*psbI* and *trnH-psbA*) and their flanking genes were seldom identified as MTPTs.

The MTPT distribution was coincident with the taxonomical groupings based on mitochondrial genes for the 81 plant species. In addition, the same pattern of MTPT distribution was identified at the family or genus level, with some exceptions. Certain species, such as two *Corchorus* species and *Capsicum annuum*, showed unique, highly abundant MTPT patterns that were extremely different from those of closely related species (denoted with arrowheads on the phylogenetic tree in Fig. 2 and Supplementary Note S7). MTPTs corresponded to 5.7% and 33.1% of the plastid genome in the mitochondrial genomes of *S. lycopersicum* and *C. annuum*, respectively, indicating that there has been recent additional plastid–mitochondrial genome flux in *C. annuum* (Supplementary Figs. 3 and 4**)**.

### DNA barcode markers based on inter- and intra-species plastid polymorphism

**Figure 3:**
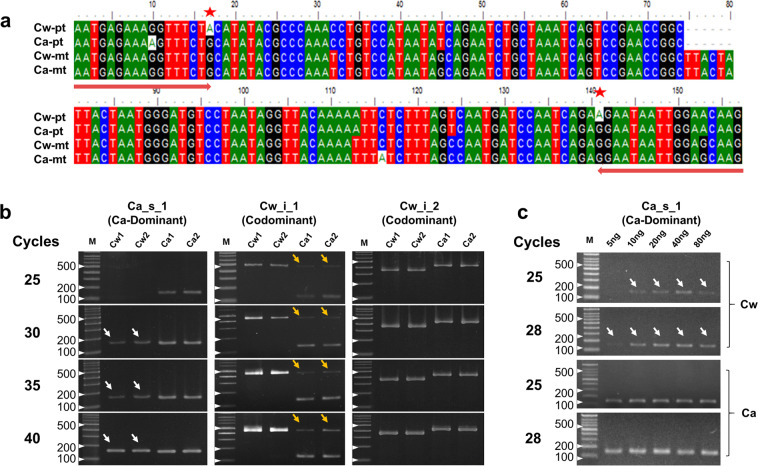
DNA marker paradox derived from MTPTs. (**a)** Amplicons of a Ca-specific marker (Ca_s_1) designed from the *matK* region and its counterpart MTPTs in *C. wilfordii* (Cw) and *C. auriculatum* (Ca). Pt, plastid genome; mt, MTPT segment in the mitochondrial genome. Primer regions and target SNPs used for authentication of Cw and Ca are marked with red arrows and red stars, respectively. **(b)** Electrophoresis of PCR products from three authentication markers for *Cynanchum* species after different numbers of PCR cycles. Ca_s_1 is a Ca dominant marker, and Cw_i_1 and Cw_i_2 are co-dominant markers. Two accessions each of Cw and Ca were used. **(c)** Results from electrophoresis of PCR products obtained from Cw and Ca samples using the Ca-specific marker Ca_s_1 with different amounts of template DNA and different numbers of PCR cycles. MTPT-derived PCR products are denoted by white and yellow arrowheads in Cw and Ca, respectively.

We identified polymorphic sites from the two *Cynanchum* species by performing pairwise alignment of the plastid genome sequences of both plants (Supplementary Table 5). Based on this information, we developed 12 DNA markers, including seven SNPs and five InDels, for the authentication of each species (Supplementary Table 6). We inspected three of these markers under different PCR conditions and found that two of them target the polymorphic plastid regions that are homologous to MTPT counterparts in the mitochondrial genomes of both species, while the third targets a polymorphic plastid region that has no MTPT counterpart (Fig. 3). The first marker is a Ca-specific marker derived from a SNP associated with *matK* that is currently used to detect Ca contamination in Cw products under obligate regulation by the Ministry of Food and Drug Safety of Korea^39,43^ (Fig. 3a). The second is based on codominant primers targeting a 348-bp InDel polymorphism located in the intergenic spacer (IGS) region between *rps2* and *rpoC2*, which produce a 481-bp band for Cw and a 135-bp band for Ca (Supplementary Fig. 2a). The third marker is also based on codominant primers, in this case targeting the IGS region between *rpoB* and *trnC-GCA* and producing a 347-bp and 428-bp band for Cw and Ca, respectively. All three markers showed the expected genotype for the plastid genomes of both species using a moderate number of PCR amplification cycles (less than 25). However, for the first and second markers, unexpected bands were detected when we increased the number of PCR cycles or the amount of template DNA (arrowheads in Fig. 3b,c). These bands were also amplified from all the 27 Cw collections in Korea (Supplementary Fig. 5a). These unexpected bands were derived from MTPT targets. The MTPTs of both species are almost identical both to each other and to the *matK* gene of the Ca plastid (Fig. 3a and Supplementary Fig. 2b). Furthermore, read mapping of WGS indicated that 88% and 12% of NGS reads were derived from the plastid and mitochondrial genomes, respectively (Supplementary Fig. 5 and Table 2**)**. These numbers might represent the copy numbers of the plastid and mitochondrial genomes within a cell and might explain the different amounts of PCR products, i.e., the appearance of weaker bands derived from MTPTs compared to intense bands for the Ca_s_1 marker and Cw_i_1 marker in Cw and Ca, respectively (Fig. 3b,c and Supplementary Note 2). Finally, we designed nine additional DNA markers: three InDel and six SNP targets from plastids without MTPT homologs. All nine markers showed clear authentication of Cw and Ca without any noise, even following numerous PCR cycles, such as the third marker in Fig. 3b (Supplementary Fig. 6).

**Table 2:** Estimated proportions of NGS reads for the plastid and mitochondrial genomes of Cw and Ca.

Plants	Total no. of reads	Total NGS data (Gbp)	Read depth at SNP sites^†^			
			Forward primer		Reverse primer	
			Pt* origin	Mt* origin	Pt* origin	Mt* origin
*C. wilfordii*	3,064,122	0.7	1045 (A)	141 (G)	1148 (A)	145 (G)
			88%	12%	88%	12%
*C. auriculatum*	3,350,936	1.0	1889 (G)	297 (G)	1833 (G)	246 (G)
			86%	14%	88%	12%

^*^Pt, Plastid; Mt, Mitochondria;

^†^The genotype of each position is shown in parentheses, ‘A’ for adenine and ‘G’ for guanine. The proportion was calculated based on read depth for the polymorphic sites between the plastid and counterpart MTPT (Supplementary Fig. 5). The plastid and mitochondrial genome copy numbers are estimated to be 1,241 and 170 copies, respectively, in a somatic cell (2n).

**Figure 4:**
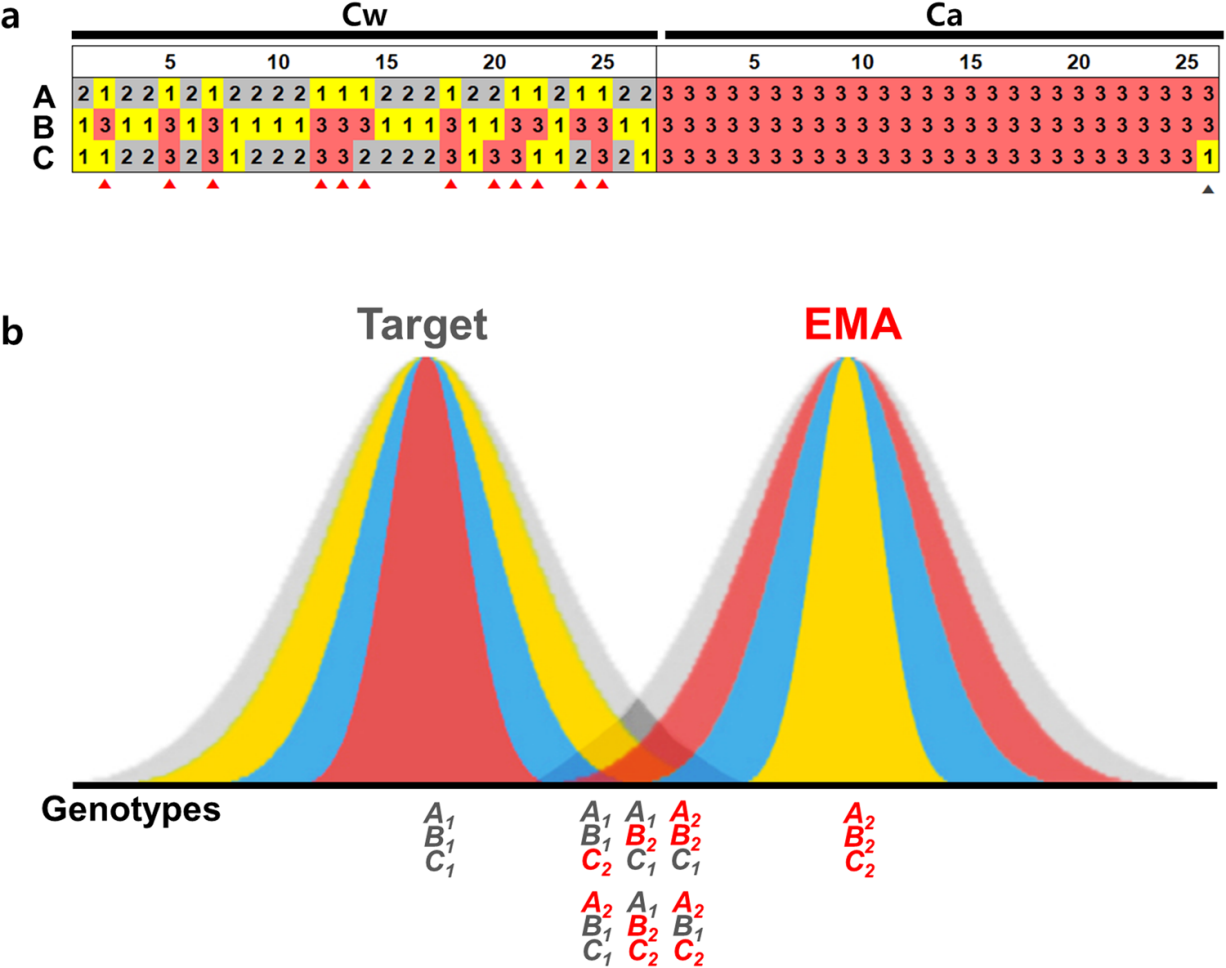
Intraspecies plastid diversity and DNA barcoding paradox. (**a**) Genotypic data for three markers across 27 Cw and 26 Ca collections. The Cw collections showing the Ca genotype and Ca collections showing the Cw genotype based on one of the three are denoted with red and gray arrowheads, respectively. A, B and C indicate markers Cw_i_6, Cw_i_7 and Cw_i_8, respectively (Supplementary Figs 5b, 8, and Table 6). (**b**) Hypothetical distribution of natural genetic diversity for target and related species. Putative genotypes for three loci are shown under the graphs. Gray represents the putative distribution of the natural population. Blue represents the putative diversity range based on a single marker. Red and yellow represent diversity ranges based on multiple markers. Diversity range differentiates how we define the species for each individual showing different genotypes. Yellow indicates the genotype range for the ‘positive selection method’, which defines individuals as genuine species based on any of the multiple positive markers. Red indicates the genotype range for the ‘negative selection method’, which defines individuals as counterfeit based on any of the negative markers.

We assembled four more plastid genomes from different Cw collections and identified 11 targets, 6 SNPs and 5 InDel regions, showing intraspecies diversity among the five plastid genomes (Supplementary Fig. 8). We designed three intra-specific polymorphic markers from the InDel targets and inspected the 27 Cw and 26 Ca collections (Fig. 4a and Supplementary Fig. 5b and Table 6). The three markers showed 2–3 haplotypes based on the copy numbers of tandem repeats. When we inspected the genotypes of 27 Cw collections using these markers, 12 showed the same genotype with Ca from at least one of the three markers.

## Discussion

Plant mitochondrial genomes are extremely large, with sizes varying from 200 to 2000 kb (Supplementary Fig. 9), i.e., 15–125 times larger than the conserved 16 kb animal mitochondrial genomes. Horizontal genome flux between the plastid, mitochondrial and nuclear genomes is an established but underappreciated phenomenon in plants. *Cynanchum* species harbor a single type of plastid genome but multi-chromosomal mitochondrial genomes, a type of structure that in other plant species is known to be derived from tandem repeat-mediated recombination^44–46^. Here, in a study of 81 angiosperm mitochondrial genomes, we detected varying degrees of plastid genome flux, with additional lineage-specific patterns, indicating that frequent plastid genome flux is quite common in angiosperms. Intriguingly, a very recent horizontal plastid genome transfer was detected in the mitochondrial genomes of some species, including *C. annuum*, indicating that plastid flux into the mitochondrial genome occurs more frequently during plant evolution^7,9,11,47–50^ (Supplementary Fig. 4). The *C. annuum* mitochondrial genome may have evolved independently through more than one period of genome flux after its divergence from the genus *Solanum*. The occurrence of multiple MTPT integration events has been inferred in several other plant lineages^19,21^^–^^24^. Furthermore, since MTPT signals gradually disappear over evolutionary time due to the accumulation of mutations, the MTPTs of *C. annuum* may reflect recent transfers, which is also in accordance with the species’ evolutionary history. Therefore, taking into account the case of *C. annuum*, it appears that the abundant, intact MTPT fragments in several species may result from multiple, recent species-specific horizontal plastid genome flux events^7,48^.

Such dynamic plastid–mitochondrial genome flux might have contributed to the diversification of the mitochondrial genome structure by contributing 2–75 kb MTPTs in 81 plants. MTPT levels and mitochondrial genome size are correlated (Supplementary Fig. 9). Plastid DNA copy numbers vary in different species and in different plant tissues. The plastid genome copy number declined rapidly, from 600 copies to fewer than 100 copies, during 5 days of dark treatment^51^. Plastid DNA is degraded by the organelle exonuclease DPD1 and contributes to phosphorus relocation^51^. When plastid DNA is not completely degraded, the abundant fragments of plastid DNA can occasionally be horizontally transferred into other cellular genomes via a mechanism involving the double-strand break repair system of the plant genome^52^.

To date, only 81 plant mitochondrial genome sequences have been reported, although thousands of plastid genomes have been characterized. This discrepancy is due to the complex mitochondrial genome structure, with an abundance of MTPTs, size variance and a high frequency of recombination. The current findings indicate that mitochondrial genomes, including MTPTs, exhibit low nucleotide substitution rates, showing more than three times slower evolution compared to plastid genomes^53^ (Fig. 1c). Importantly, we demonstrated that plant barcoding targets are found in MTPTs (Fig. 2a**)** at meaningful frequencies in a diverse array of plants. Overall, we have uncovered a notable shortcoming of the common practice of barcode-based authentication of plant products by demonstrating that a wide range of horizontal transfer events involve MTPTs, which are likely to result in the co-amplification of unexpected bands (Fig. 3b,c and Supplementary Fig 5a). These promiscuous DNAs could affect molecular taxonomy by causing mis-positioning of species if the plastid DNA sequences used were confused with those of MTPTs^8^.

EMA is estimated to have cost the herbal supplement industry 10–15 billion dollars per year since 2010^15^. The impact of mis-authentication caused by the DNA marker paradox could have severe negative effects not only on the industry but also on all parties involved in the herbal supplement industry, from farmers to consumers and beyond, as demonstrated by the involvement of the New York State Attorney General’s Office and the Korean Supreme Court in regard to *Cynanchum* products in 2015–2017. To avoid the incorrect application of DNA markers and thus to escape the DNA marker paradox, it is desirable to use multiple markers derived from different loci, which can credibly distinguish EMA from target plant products such as species-specific markers **(**Fig. S7, Supplementary Table 6**)** as the suggestion for DNA barcoding (Group *et al*.^42^). How^42^ the number of markers required to detect EMA does not need to be as high as that used in forensics to distinguish individuals of the same species (*Homo sapiens*).

Unlike major crops, herbal medicinal plants still have a wide range of natural intraspecies diversity because diverse collections are wildcrafted or undomesticated cultivated^1^^–^^4^. Therefore, there always be the possibility of false decision for the off-type individuals from molecular marker application as in the previous adulteration issue of Cw and Ca **(**Supplementary Fig. 5a**)**. We inspected the previously reported authentication markers for Cw and Ca in Korea. The *psbA-trnH* marker^37^ identified both species almost correctly, but make a few case of false- negative (decide Ca plants as Ca-negative) detection that provide Cw genotype for a Ca collection. Another marker derived from SNPs in *trnL* loop^38^ made false- positive (decide Cw plants as Ca-positive) errors for 2 of 27 Cw collections (Supplementary Fig 5a**)**. Additionally, we inspected the haplotype diversity of Cw and Ca for 3 of the 11 intraspecies polymorphic sites revealed from a comparison of the five plastid genomes of Cw (Fig. 4a and Supplementary Fig. 5b, 8 and Table 5). Based on the genotypes for the three markers, 27 Cw collections were classified into 5 groups, unlike Ca individuals, which are derived from a few Chinese collections and thus showed narrow genetic diversity. Notably, the application of the markers would place 12 of the 27 Cw collections as Ca and 1 of the 26 Ca collections as Cw if genotyping were based on data from individual negative genotypes. These findings emphasize the importance of marker choice and relying on more than one marker for authentication purposes.

To define the difficulty more generally, if the target species shows homogeneous genotypes for three loci, denoted *A*, *B* and *C*, the *A*_1_*B*_1_*C*_1_ genotype can be clearly distinguished from the *A*_2_*B*_2_*C*_2_ genotype. However, we found that many wild accessions have heterogeneous genotypes, such as *A*_1_*B*_1_*C*_2_ or *A*_2_*B*_1_*C*_2_, as a result of intraspecies plastid genome diversity (Fig. 4) or plastid–mitochondrial genome flux (Fig. 3)^1,54^. If we were to focus only on the negative markers and assume that the heterogeneous genotypes represented counterfeit products (i.e., the negative detection method), the chance of detecting the counterfeits (the detection power) would be increased, but the possibility of a decision error that defines the genuine product as fake (the rate of false-positive, type I error) would also be increased. By contrast, if we were to focus on the positive markers and to assume that the heterogeneous genotypes represent the target product (i.e., the positive detection method), we could expect the opposite result: we would accept diverse genotypes as representing the target species and thereby reduce false-positive, but we would also reduce the detection power (Fig. 4b). Our analysis showed that 12 of the 27 Cw collections would be mis-authenticated if we applied the negative detection method. Conversely, the positive detection method would define all 27 Cw collections as genuine, and 1 of 26 Ca collections would be mis-authenticated as Cw (Fig. 4a). If we applied the negative detection method to increase the detection power of an assay, the false-positive error rate would be increased; likewise, if we applied the positive detection method to reduce the false-positive error rate, the detection power would be reduced.

In general, herbal plant resources are underdeveloped, and thus their genetic diversity is currently unknown or underestimated. Given this necessary balancing act, as long as the counterfeit plant is nontoxic, we propose that the positive detection method should be applied to reduce false-positive errors, even if the detection power is reduced and the false-negative rate (Type II error) is thereby increased. This approach could reduce unforced errors resulting in the sanctioning of genuine products and thereby protect the industry. However, in cases where there are safety concerns, such as toxicity associated with the EMA counterpart, the negative detection method should be applied to maximize the detection power even at the cost of more false-positive errors, because trace amounts of adulterant could pose a threat to both consumer health and the long-term success of the industry.

The raw materials for most herbal products are heterogeneous because they are collected from natural habitats or cultivated from wild collections. The breeding of superior cultivars and the establishment of quality management systems should be encouraged to produce consistent functional foods. The production of functional foods from specific cultivars managed from seed to final products with traceability will benefit from the development of scientifically well-supported cultivar-specific markers and minimize the damage from EMA, thus promoting the growth of the functional food and herbal supplement industry.

## Materials and Methods

### Plant samples and DNA extraction

The 27 Cw materials for were collected from 6 different local-farming areas in Korea, and the 26 Ca materials were provided by the Rural Development Administration (RDA). Total genomic DNA was extracted from the samples using modified CTAB methods^55^ for NGS analysis from 5 Cw samples and 1 Ca sample. For marker application, total genomic DNA was extracted from the samples using a Genomic Plus DNA Prep Kit (Inclone Biotech, Korea) following the manufacturer’s instructions.

### Assembly of plastid and mitochondrial genomes

We produced Illumina platform WGS Paired-End data using genomic DNA from individual *Cynanchum wilfordii* (Cw) and *Cynanchum auriculatum* (Ca) plants. We assembled the plastid genome sequence^40,41^ (GenBank accession numbers NC_029459 and NC_029460) and mitochondrial genome sequence (GenBank accession numbers MH931257, MH931258, MH931259 and MH931260) using the WGS datasets. After trimming of low-quality reads, assembly was performed using CLC genome assembler (ver_4.01). The mitochondria-related contigs were extracted from the assembled contigs via comparison with the mitochondrial sequence of *A. syriaca* (NC_022796) using MUMmer^56^. Contigs showing over than 99% homology and overlap with the plastid genome were removed and the remaining mitochondrial DNA contigs were combined for each type of mitochondrial genome and validated by mapping of raw reads using the dnaLCW method^2,3^. Additionally, four independent Cw collections were sequenced on the MiSeq platform (Illumina, San Diego, CA), and their plastid genomes were also assembled following the methods described above (MK182385, MK182386, MK182387, MK182388).

### Identification of MTPTs in the mitochondrial genome

To detect MTPTs in plant mitochondrial genome sequences, we compared 81 mitochondrial genome sequences, including the newly assembled mitochondrial genomes of Cw and Ca and 79 published plant mitochondrial genomes **(**Supplementary Table 1) retrieved from the NCBI database. Each mitochondrial genome sequence was compared with the plastid genome sequence of *A. thaliana* (NC_000932) using BLASTN^57^ based on more than 70% identity from at least 30 bp strands with an expectation value of 1E-10. The regions of the plastid genome matching transfer RNA (tRNA) or ribosomal RNA (rRNA) were ignored and excluded from this study because tRNA and rRNA are conserved in both organelle genomes.

### Plastid marker design and PCR amplification

The plastid sequences of the *Cynanchum* species were aligned using MAFFT^58^. After identifying polymorphic regions by pairwise alignment of the Cw and Ca chloroplast genomes, we mapped each raw NGS read to the corresponding chloroplast genome to detect promiscuous regions including MTPT. SNPs or InDels showing heterogeneous genotypes for more than 3% of each read depth were eliminated. Primers for codominant markers and high-resolution melting curve (HRM) analysis were designed using Primer-BLAST^59^. We selected InDel polymorphic regions whose length was greater than 20 bp and converted these regions to the codominant marker. In the case of SNPs, we filtered out adjacent SNPs less than 150 bp long and designed primers flanking candidate SNPs with lengths ranging from 100 to 150 bp.

PCR was conducted using the following cycling program: pre-amplification at 95 °C for 5 min; 25–40 cycles of denaturation at 95 °C for 30S ec, annealing at 58 °C for 30 sec, and elongation at 72 °C for 30 sec; and a final elongation at 72 °C for 5 min. The mixture consisted of a total volume of 25 μL prepared using an Inclone^TM^ Taq DNA Polymerase Kit (Inclone, South Korea) with 5–80 ng of template DNA, 1× PCR reaction buffer, 0.2 mM of each dNTP, 0.2 pmol of each primer (Bioneer, South Korea) and 0.4 units of Taq DNA polymerase. Ca-specific markers were amplified as described previously^38^. High-resolution melting-curve (HRM) analysis was performed via detection on a LightCycler 480 Real-time PCR Machine (Roche Applied Science, Indianapolis, IN, United States) under the following conditions: pre-amplification at 95 °C for 5 min followed by 45 cycles of 95 °C for 30 sec, 58 °C for 30 sec, and 72 °C for 30 sec. The reaction mixture (20 μL) contained 20 ng of template DNA, 0.5 pmol of each primer (Bioneer, South Korea) and a 1× Pre-mix of RealHelix^TM^ Premier qPCR Kit (Nanohelix, South Korea). The uncropped gel images are shown in Supplementary Fig. 10.

KASP (Kompetitive allele-specific PCR) markers were designed from the same SNP positions used for HRM analysis with Kraken software (LGC Genomics, Hoddeson, UK). Thermocycling and endpoint genotyping for the KASP assays were applied to the Cw and Ca populations using a Roche LC480 (Roche Applied Science, Indianapolis, IN, United States) following the manufacturer’s instructions in a total reaction volume of 10 μL containing 100 ng of template DNA, 0.14 μL of KASP assay mix and 5 μL of KASP reaction mix.

### Ks value calculation for genes in plastid and mitochondrial genomes

We calculated the level of synonymous substitutions per synonymous site (Ks) between homologous genes in the plastid genomes (plastid genes, PT), mitochondrial genes (MT) and MTPTs of four Apocynaceae species, Cw, Ca, *Asclepias syriaca*^9^ and *Rhazya stricta*^60^. Pairwise alignments of the coding sequences for common genes derived from each organelle were conducted using webPRANK^61^ based on translated codons. After alignment, InDel regions and stop codons were trimmed using GBLOCKS^62^, and Ks values were calculated using CODEML from the PAML package^63^. Divergence time was estimated as Ks/2λ, where λ represents the synonymous substitution rate of 2 × 10^−9^ for the plastid genomes and 0.6 × 10^−9^ for the mitochondrial genomes.

## Supplementary information

Supplementary Figures and Tables.

## Data Availability

All sequence data used in this study have been deposited in the NCBI Nucleotide Database. The accession numbers of sequence data for the mitochondrial genomes are listed in Table S1. For the plastid genome, *C. wilfordii* (NC_029459, MK182385, MK182386, MK182387, MK182388), *C. auriculatum* (NC_029460) and *A thaliana* (NC_000932) were used.
