# Author Correction: Mitochondrial plastid DNA can cause DNA barcoding paradox in plants

**DOI:** 10.1038/s41598-021-89266-5

**Published:** 2021-04-29

**Authors:** Hyun-Seung Park, Murukarthick Jayakodi, Sae Hyun Lee, Jae-Hyeon Jeon, Hyun-Oh Lee, Jee Young Park, Byeong Cheol Moon, Chang-Kug Kim, Rod A. Wing, Steven G. Newmaster, Ji Yeon Kim, Tae-Jin Yang

**Affiliations:** 1 Department of Plant Science, Plant Genomics and Breeding Institute, and Research Institute of Agriculture and Life Sciences, College of Agriculture and Life Sciences, Seoul National University, Seoul, 08826 Republic of Korea; 2 Phyzen Genomics Institute, Seongnam, 13558 Korea; 3 Herbal Medicine Research Division, Korea Institute of Oriental Medicine, 1672 Yuseong-daero, Yuseong-gu, Daejeon, 34054 Republic of Korea; 4 Genomics Division, National Institute of Agricultural Sciences, Jeonju, 54874 Republic of Korea; 5 Arizona Genomics Institute, School of Plant Sciences, The University of Arizona, Tucson, AZ USA; 6 NHP Research Alliance, College of Biological Sciences, University of Guelph, Guelph, ON Canada; 7 Department of Food Science and Technology, Seoul National University of Science and Technology, Seoul, 01811 Korea

Correction to: *Scientific Reports* 10.1038/s41598-020-63233-y, published online 09 April 2020

This Article contains a typographical error in the Introduction section where,

“This adulteration claim was refuted, and the case was acquitted by the Korean Supreme Court due to the lack of properly validated scientific methods and the use of only one or two plastid DNA markers, which were inadequate for identifying these ingredients, as these barcodes could not discriminate these two closely related plant species.”

should read:

“This adulteration claim was refuted, and the case was acquitted by the Korean Prosecution Service due to the lack of properly validated scientific methods and the use of only one or two plastid DNA markers, which were inadequate for identifying these ingredients, as these barcodes could not discriminate these two closely related plant species.”

In the Discussion section,

“The impact of mis-authentication caused by the DNA marker paradox could have severe negative effects not only on the industry but also on all parties involved in the herbal supplement industry, from farmers to consumers and beyond, as demonstrated by the involvement of the New York State Attorney General’s Office and the Korean Supreme Court in regard to Cynanchum products in 2015–2017.”

should read:

“The impact of mis-authentication caused by the DNA marker paradox could have severe negative effects not only on the industry but also on all parties involved in the herbal supplement industry, from farmers to consumers and beyond, as demonstrated by the involvement of the New York State Attorney General’s Office and the Korean Prosecution Service in regard to Cynanchum products in 2015–2017.”

