# Supplementary material for: Mitochondrial plastid DNA can cause DNA barcoding paradox in plants
Source: Sci Rep. 2020 Apr 9;10:6112. doi: 10.1038/s41598-020-63233-y (PMC7145815; doi:10.1038/s41598-020-63233-y)
Supplement: Supplementary file 1 — Supplementary Figures and Tables. [file 41598_2020_63233_MOESM1_ESM.docx]

**Mitochondrial plastid DNA can cause DNA barcoding paradox in plants**

Hyun-Seung Park^1^, Murukarthick Jayakodi^1^, Sae Hyun Lee^1^, Jae-Hyeon Jeon^1^, Hyun-Oh Lee^2^, Jee Young Park^1^, Byeong Cheol Moon^3^, Chang-Kug Kim^4^, Rod A. Wing^5^, Steven G. Newmaster^6^, Ji Yeon Kim^7^ Tae-Jin Yang^1*^

Hyun-Seung Park (fgenesis@snu.ac.kr)

^1^Research Institute of Agriculture and Life Sciences, College of Agriculture and Life Sciences, Seoul National University, Seoul 08826, Republic of Korea.

Murukarthick Jayakodi (starmuru88@gmail.com)

^1^Research Institute of Agriculture and Life Sciences, College of Agriculture and Life Sciences, Seoul National University, Seoul 08826, Republic of Korea.

Sae Hyun Lee (whyskyisgray@naver.com)

^1^Research Institute of Agriculture and Life Sciences, College of Agriculture and Life Sciences, Seoul National University, Seoul 08826, Republic of Korea.

Jae-Hyeon Jeon (wwjun436@gmail.com)

^1^Research Institute of Agriculture and Life Sciences, College of Agriculture and Life Sciences, Seoul National University, Seoul 08826, Republic of Korea.

Hyun-Oh Lee (dlgusdh88@phyzen.com)

^2^Phyzen Genomics Institute, Seongnam 13558, Korea.

Jee Young Park (jypark74@snu.ac.kr)

^1^Research Institute of Agriculture and Life Sciences, College of Agriculture and Life Sciences, Seoul National University, Seoul 08826, Republic of Korea.

Byeong Cheol Moon (bcmoon@kiom.re.kr)

^3^Herbal Medicine Research Division, Korea Institute of Oriental Medicine, 1672 Yuseong-daero, Yuseong-gu, Daejeon 34054, Republic of Korea

Chang-Kug Kim (chang@korea.kr)

^4^Genomics Division, National Institute of Agricultural Sciences, Jeonju, 54874, Republic of Korea

Rod A Wing (rwing@ag.arizona.edu)

^5^Arizona Genomics Institute, School of Plant Sciences, The University of Arizona, Tucson, AZ, USA

Steven G Newmaster (snewmast@uoguelph.ca)

^6^NHP Research Alliance, College of Biological Sciences, University of Guelph, Guelph, Ontario, Canada.

Ji Yeon Kim (jiyeonk@seoultech.ac.kr)

^7^Department of Food Science and Technology, Seoul National University of Science and Technology, Seoul 01811, Korea

Tae-Jin Yang (tjyang@snu.ac.kr)

^1^Research Institute of Agriculture and Life Sciences, College of Agriculture and Life Sciences, Seoul National University, Seoul 08826, Republic of Korea.

*Correspondence to: Tae-Jin Yang ([tjyang@snu.ac.kr](mailto:tjyang@snu.ac.kr))

**Supplementary Information**

**Supplementary Note 1. Plastid genome flux into the mitochondrial genome in angiosperms**

Mitochondrial sequences of plastid origin (MTPTs) in the mitochondrial genome sequences of 81 flowering plants showed taxonomical-lineage-specific patterns. We conducted phylogenetic analysis based on the conserved mitochondrial gene, *matR*. The taxonomic relationship and MTPT distribution patterns showed a mixture of common and unique patterns for each family or genus level (**Fig. 2**). Frequent MTPT insertions have been found in most monocots and several lineages of eudicots, especially the Cucurbitales, Lamiales and Gentianales^17,21,24-26,44,45^. In the monocot order Poales, *rpl2* and the region from *atpE* to *rbcL* were shared, with some exceptions. In particular, the three *Zea* species were distinguished from other Poales (*Oryza* and *Bambusa* species) by their shorter fragment in *psbA* and the lack of *rpoC2* and *rpl14* regions.

In the order Brassicales, MTPTs showed family-level common distribution patterns and genus-level unique patterns. The gene fragments of *psaA*, *rbcL*, *ycf2*-*trnL* and *ycf1* were shared between *Brassica* species and *Schrenkiella parvula*, but *rpoB* was only found in *Brassica*. The *Arabidopsis* genus contains only short regions of *rbcL* and *ycf1* fragments. Other species assessed belonged to different families in the Brassicales, for example *Batis maritima* and *Carica papaya*. In the order Malpighiales, the Salicaceae show abundant MTPT fragments that are not found in the Euphorbiaceae.

Some of the plants showed extremely different MTPT insertion patterns compared to closely related species. Within the order Malvales, two genera, *Corchorus* and *Gossypium*, showed very different patterns, with only a few MTPT insertions in *Gossypium* but frequent insertions in *Corchorus*. In the Solanales, extremely large MTPT insertions were detected in the mitochondrial genome of *Capsicum annuum*, and the mitochondrial genome structure of *C. annuum* was very different from those of *Solanum* species. The mitochondrial genome of *C. annuum* showed a great deal of recombination compared to those of *Solanum tuberosum* and *S. lycopersicum* (**Supplementary Fig. 3**). There were few MTPTs in *S. lycopersicum* (5.7% of the plastid genome), and most were located in regions homologous to the *C. annuum* mitochondrial genome (**Supplementary Fig. 4**), but *C. annuum* also showed additional, exceptionally large MTPTs (33.1% of the plastid genome).

**Supplementary Note 2. DNA markers based on plastid polymorphism**

We identified hundreds of polymorphic sites between *Cynanchum* *wilfordii* (Cw) and *Cynanchum* *auriculatum* (Ca) based on pairwise alignment of the plastid sequences of the two species. From this we developed 12 DNA markers, including one known and 11 novel markers, seven single-nucleotide polymorphisms (SNPs) and five insertions and deletions (InDels), to use in the evaluation of each species (**Supplementary Table 6**). Among these 12 markers, we investigated three using the same primer pairs under diverse PCR conditions.

For the first set of primers, an unexpected band was amplified from Cw, which was slightly larger than the original band from Ca. For the second set of primers, an unexpected band was detected from Ca that was similar in size but slightly smaller than the Cw–specific band. In order to trace the amplification of these bands, we repeated the PCR experiment with different numbers of PCR cycles, and we found that the unexpected bands were already present, at low intensity, starting from the 25^th^ PCR cycle and became more intense as the cycle number increased. However, the bands for the third set of markers remained identical as the number of PCR cycles increased.

To determine the origins of these unexpected bands, we inspected the amplicon sequences in both the plastid and mitochondrial genomes. The first marker was designed from the *matK* coding sequence (CDS), with adenine (A) for Cw and guanine (G) for Ca. Pairwise alignment of the *matK* target region in the plastids and MTPTs of both species revealed high sequence similarity, with 94.5% in Cw and 94.4% in Ca. The *matK* homologs in MTPTs of both species are almost identical and showed the same SNP allele in the primer target as the plastid genome of Ca (**Fig. 3a**). Based on the results obtained by mapping the complete next-generation sequencing (NGS) data from Cw to this region, 12% of NGS reads contained the mitochondrial nucleotide variant (G) at two SNP priming sites in the *matK* CDS (**Supplementary** **Fig. 2a and 5a and Table 1**). Mapping of all the NGS data from Ca revealed no sequence variation at the two SNP sites, although 12–14% of the NGS reads were derived from the mitochondrial genome, which indicates that the primers amplify both plastid and mitochondrial genome DNA (**Supplementary Fig. 5b and Table 1**).

For the second marker, the forward and reverse primer sequences were exactly the same as those in plastids and MTPTs in both species. However, the plastid amplicon of Ca showed a 346-bp deletion compared to the plastid amplicon of Cw. The MTPTs of both species were conserved and had fewer InDels than the plastid amplicon of Cw (**Supplementary Fig. 2b**). Therefore, these plastid-derived and MTPT-derived bands were co-amplified in both species. However, in Cw, they gave the appearance of a single band due to the similar sizes of the plastid and MTPT amplicons. In Ca, two unique bands were amplified, but the upper band was weaker than the lower band because the upper band was derived from an MTPT and the lower band was derived from the plastid target, which shows copy number variation within a cell (**Fig. 3b**).

For the third marker, the plastid amplicons did not show any similarity to the mitochondrial genome in either species, and they showed clear single bands whose sizes differed between the two species. We also designed three more codominant InDel markers from unique plastid genomes without MTPT homologs (**Supplementary Table 6**), and we found that all three of these markers clearly distinguished between individuals of Cw and Ca.

The other three InDel and six SNP markers were also developed from SNPs without MTPT homologs. The three InDel markers showed clear authentication via normal PCR analysis and agarose gel electrophoresis. We assessed the six SNP markers using high-resolution melting curve (HRM) analysis and the Kompetitive allele-specific PCR (KASP) method. When applied to multiple samples collected from various regions, these markers showed clear authentication of Cw and Ca individuals using any platform (**Supplementary Fig. 6**).

Supplementary Fig. 1. Mitochondrial genome maps of *C. wilfordii* *and C. auriculatum*. (a to c), Mitochondrial genome types 1, 2 and 3 for *C. wilfordii*. (d) Mitochondrial genome of *C. auriculatum*.


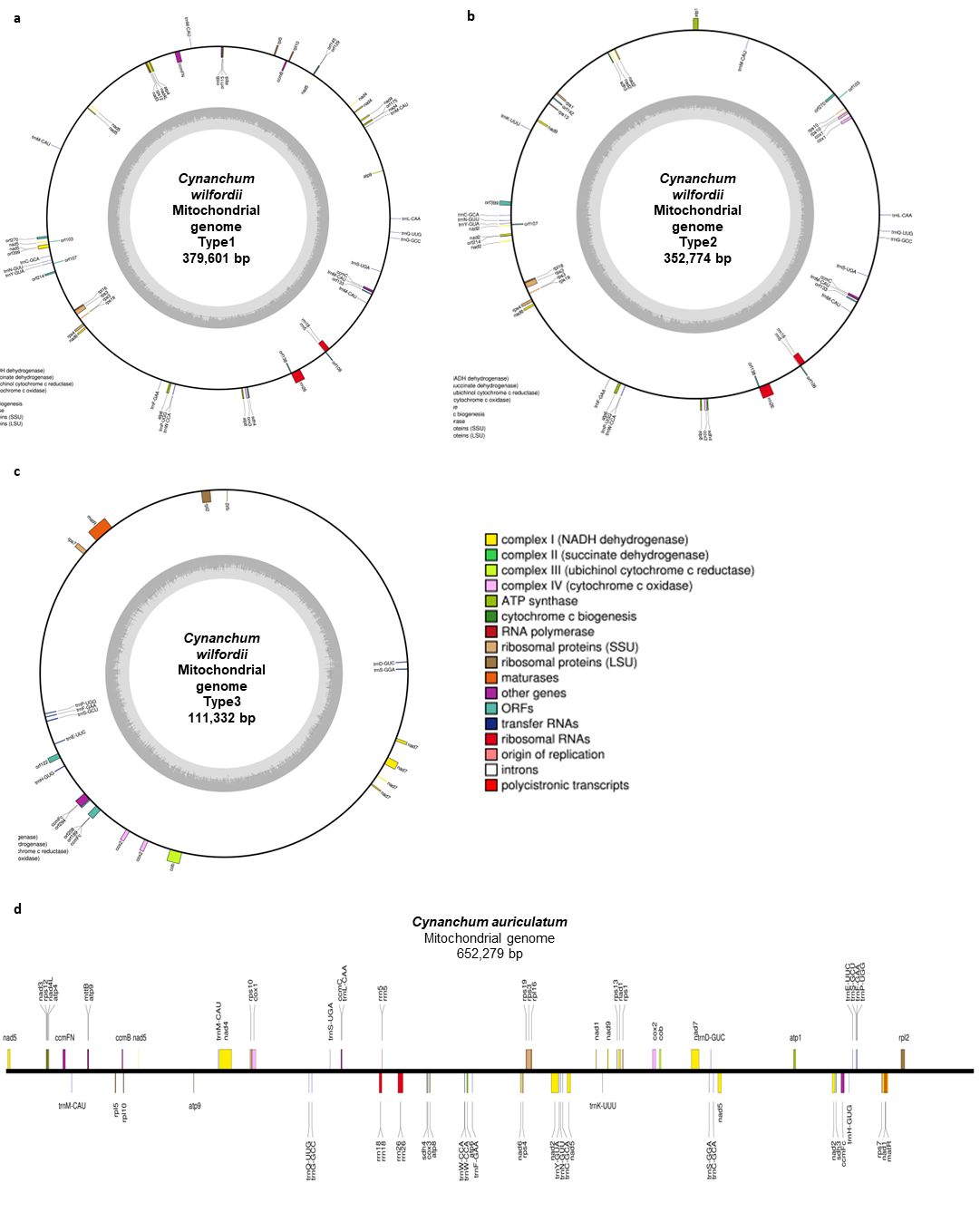


Supplementary Fig. 2. Multiple sequence alignment of two plastid targets with their counterpart MTPT targets in *C. wilfordii* (Cw) and *C. auriculatum* (Ca). (a) Multiple sequence alignment of the full sequence of the *matK* gene and counterpart MTPTs in *C. wilfordii* and *C. auriculatum* (b) Multiple sequence alignment of the amplicon of Cw_i_1 representing plastid and counterpart MTPT targets in Cw and Ca. Blue arrows indicate primers in the intergenic region of *rps2* and *rpoC2*.


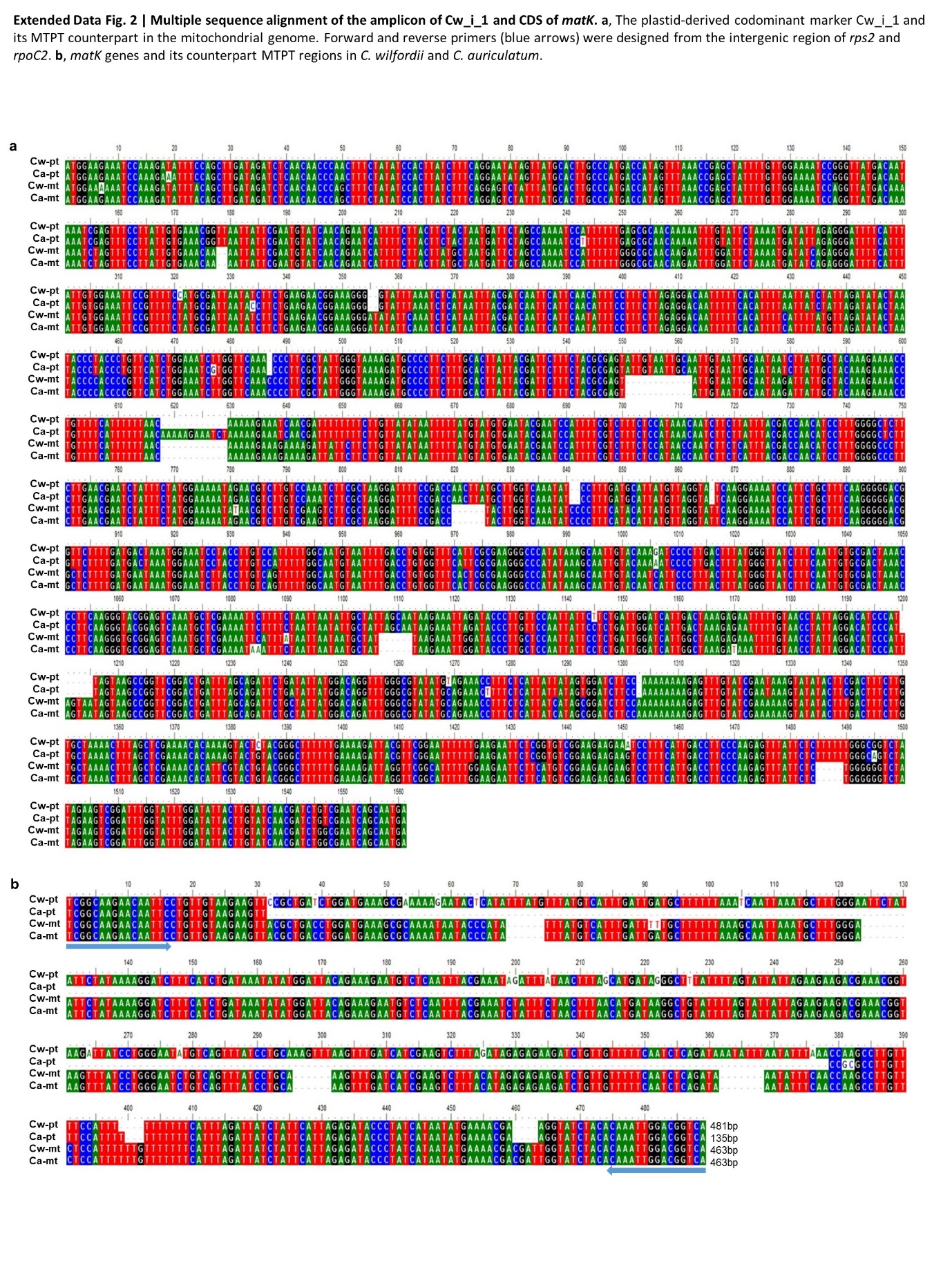


Supplementary Fig. 3. Genome-wide comparison of the mitochondrial genomes of three Solanaceae species: pepper, tomato and potato.


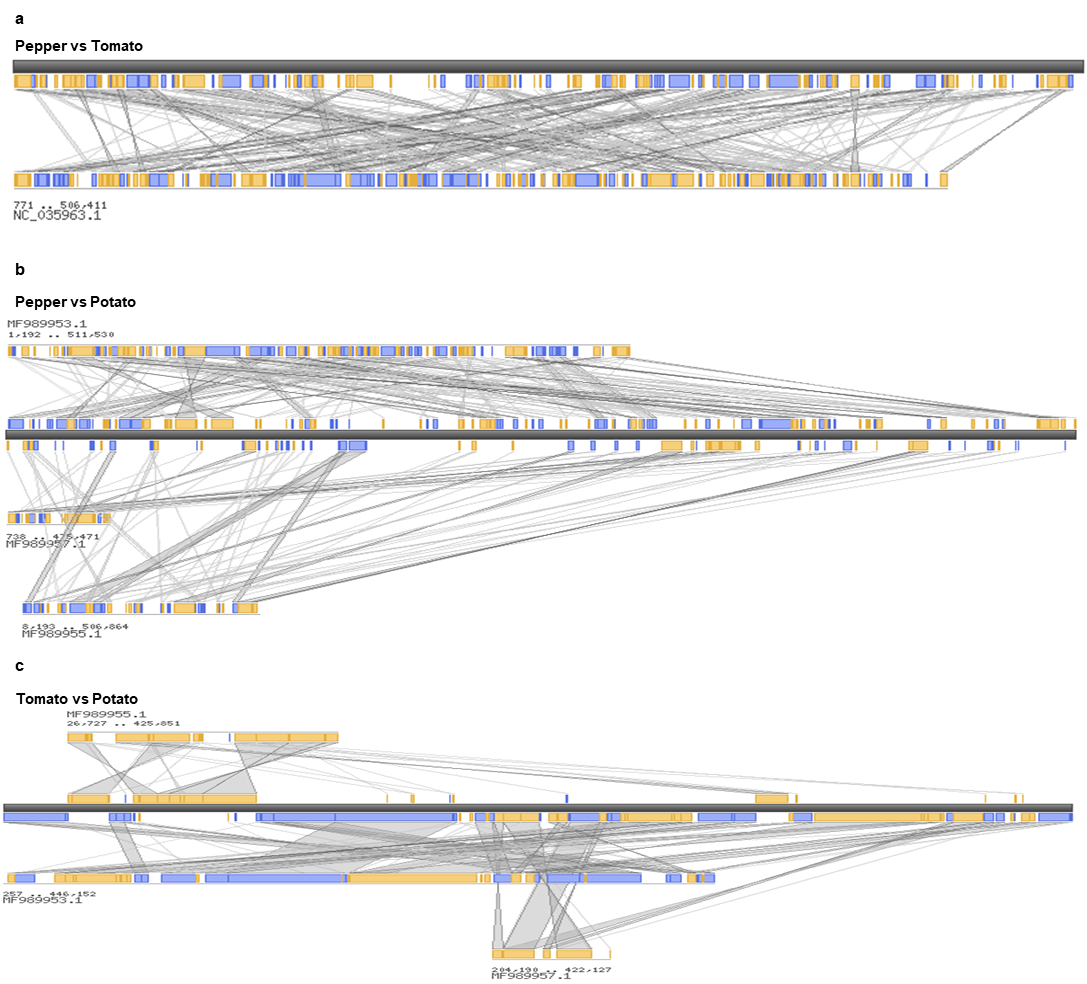


Supplementary Fig. 4. Circos plot showing MTPTs in *Capsicum annuum* and *Solanum lycopersicum.* The plastid genome sequence was compared with the mitochondrial genomes of *C. annuum* and *S. lycopersicum.* The plastid genome (top) and its MTPT counterparts are denoted by blue and red brackets for the mitochondrial genomes of *C. annuum* and *S. lycopersicum*, respectively.

**
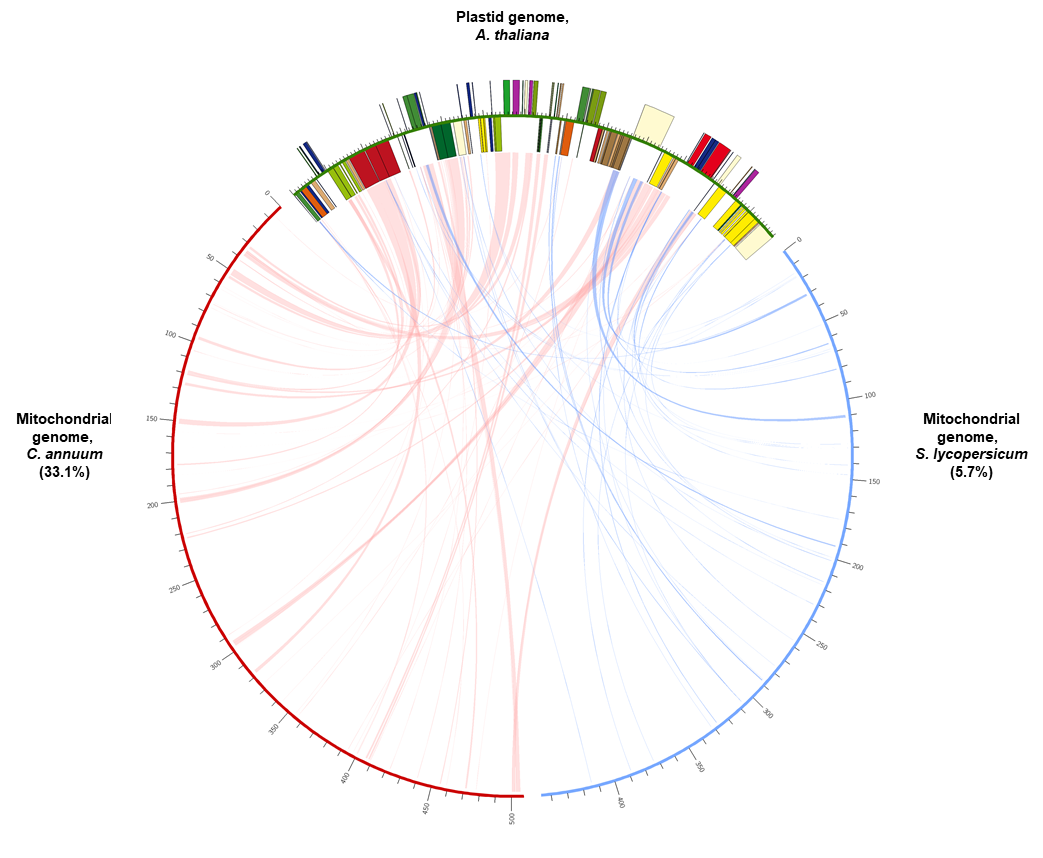
**

**Supplementary Fig. 5. Genotyping result of plastid-derived markers from multiple samples of *Cynanchum* species including 27 of *C. wilfordii* and 26 of *C. auriculatum*.** (a) Genotyping results of the previously reported markers for authentication of Cw and Ca. False-positive signals are marked with light blue stars. Ca_s_1 is derived from *matK* in Fig 3a. CwF and CaF are derived from the *trnL* (tRNA-Leu) intron region^38^. Cw_Ca_codominant is derived from intergenic region of *trnH* and *psbA*^37^. (b) Genotyping result of three InDel markers derived from intra-specific variation among *C. wilfordii*.


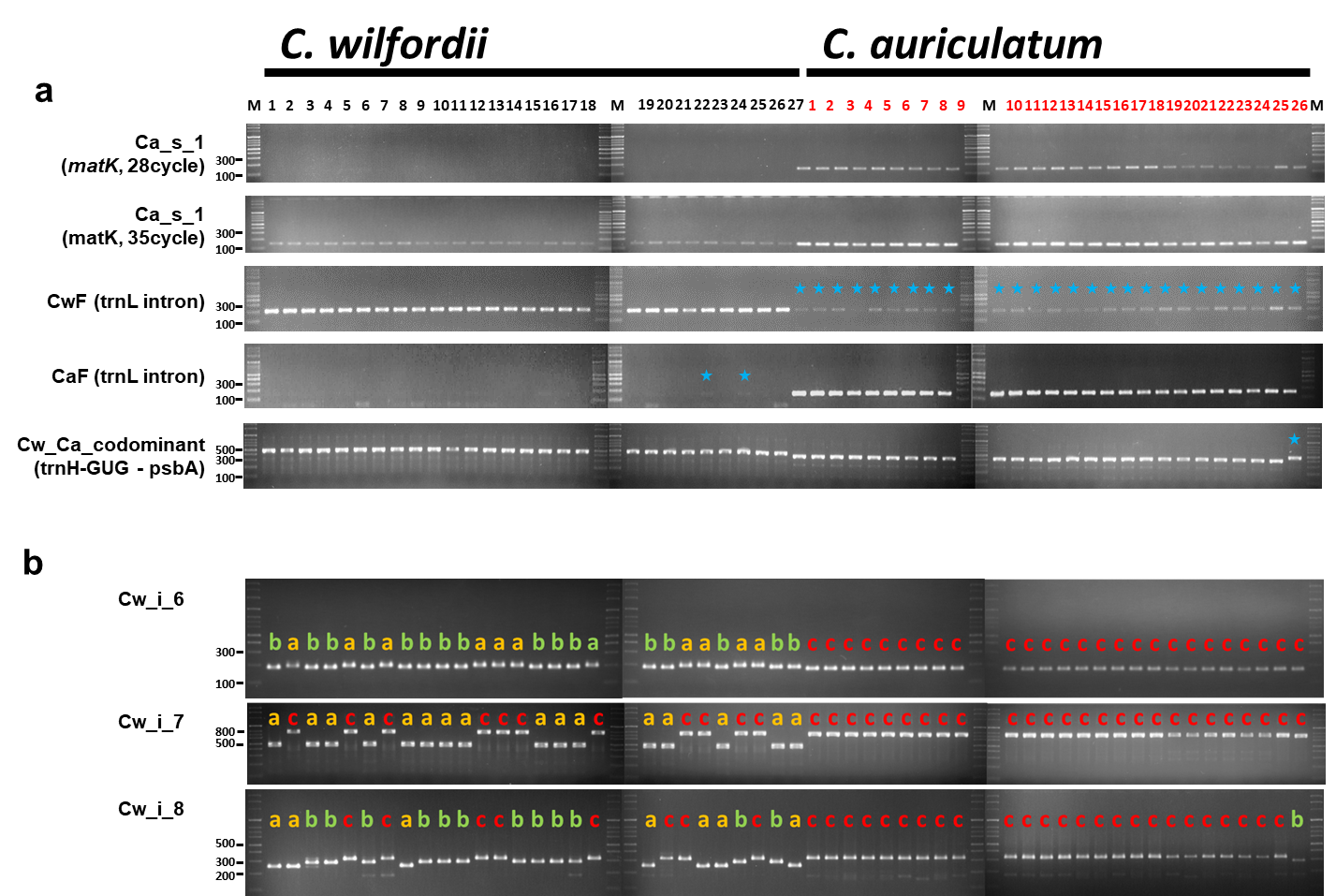


Supplementary Fig. 6. Estimated proportions of plastid (Pt) and mitochondrial (Mt) genomes using WGS of *C. wilfordii* (Cw) and *C. auriculatum* (Ca). (a and b), partial image capture for NGS read mapping on the *matK* target using WGS of Cw and Ca, respectively. Red arrows represent primer regions and red stars indicate target SNPs for discriminating Cw and Ca. The NGS reads putatively derived from the mitochondrial genome are marked with black arrows.


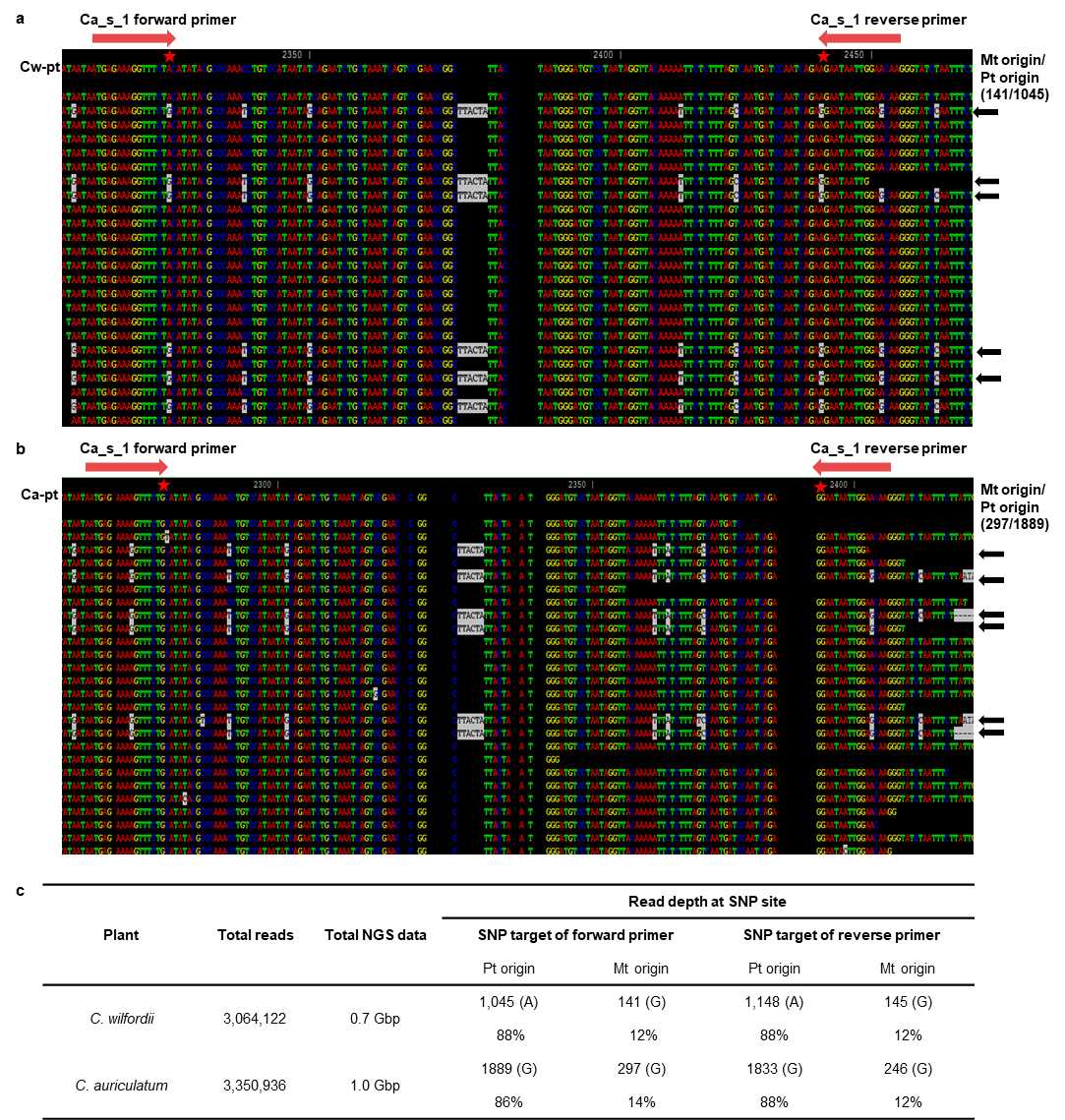


Supplementary Fig. 7. Nine additional plastid markers without MTPT counterparts for authentication of *C. wilfordii* (Cw) and *C. auriculatum* (Ca). (a) Agarose gel electrophoresis of four co-dominant markers for authentication of *Cynanchum*. (b) Genic SNPs between two *Cynanchum* species and application of HRM analysis for authentication. (c) Scatter plot of KASP markers derived from the SNPs in (b) applied to the Cw and Ca populations


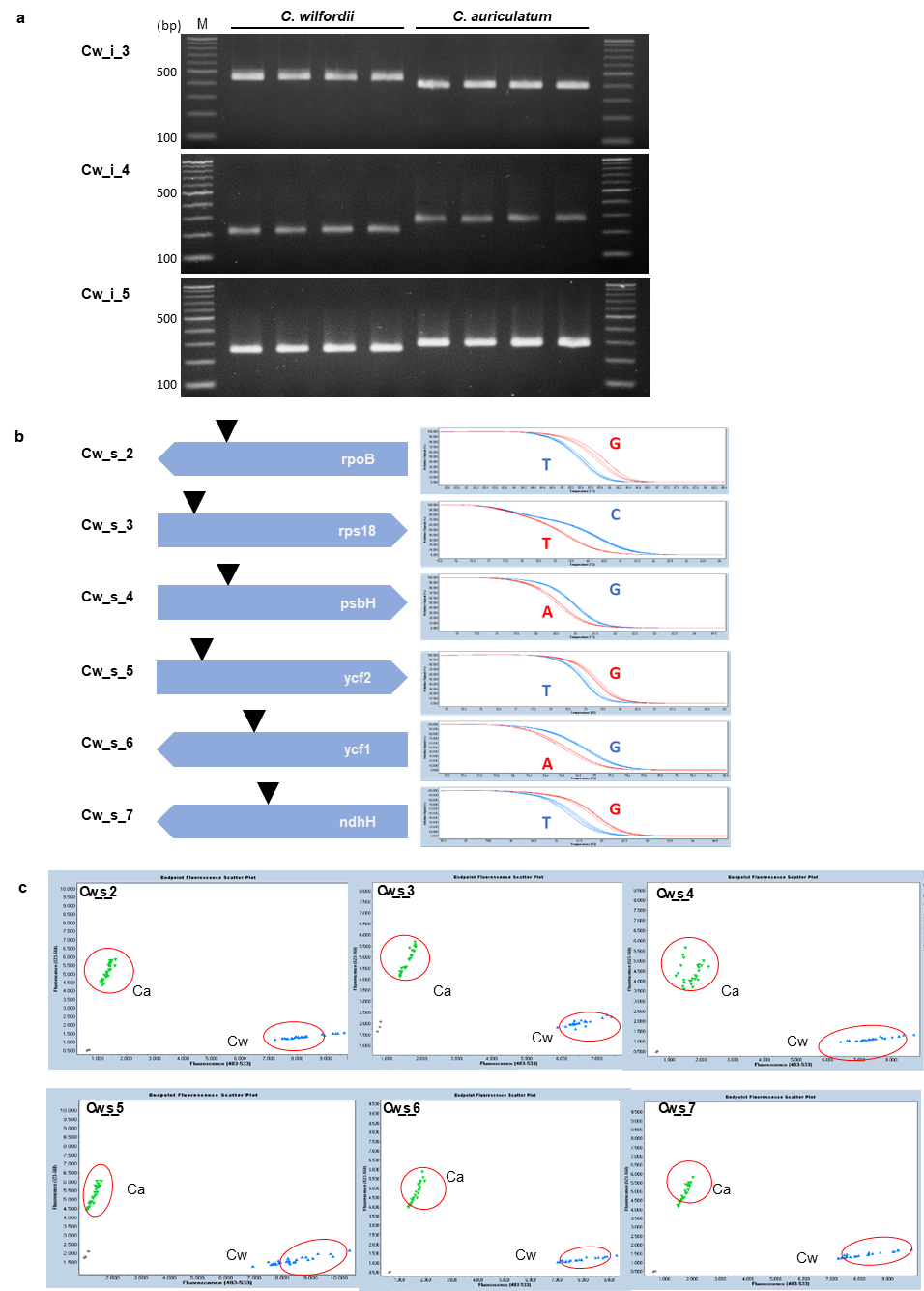


**Supplementary Fig. 8. Plastid map and intra- and inter-species polymorphism of *C. wilfordii.*** Intraspecies diversities and InDels and SNPs are denoted by red and blue arrows, respectively. Three InDel markers are denoted with marker names. Inter-species variations and InDels and SNPs between *C. wilfordii* (Cw) and *C. auriculatum* (Ca) are indicated in the inner circle by red and blue lines, respectively.

**
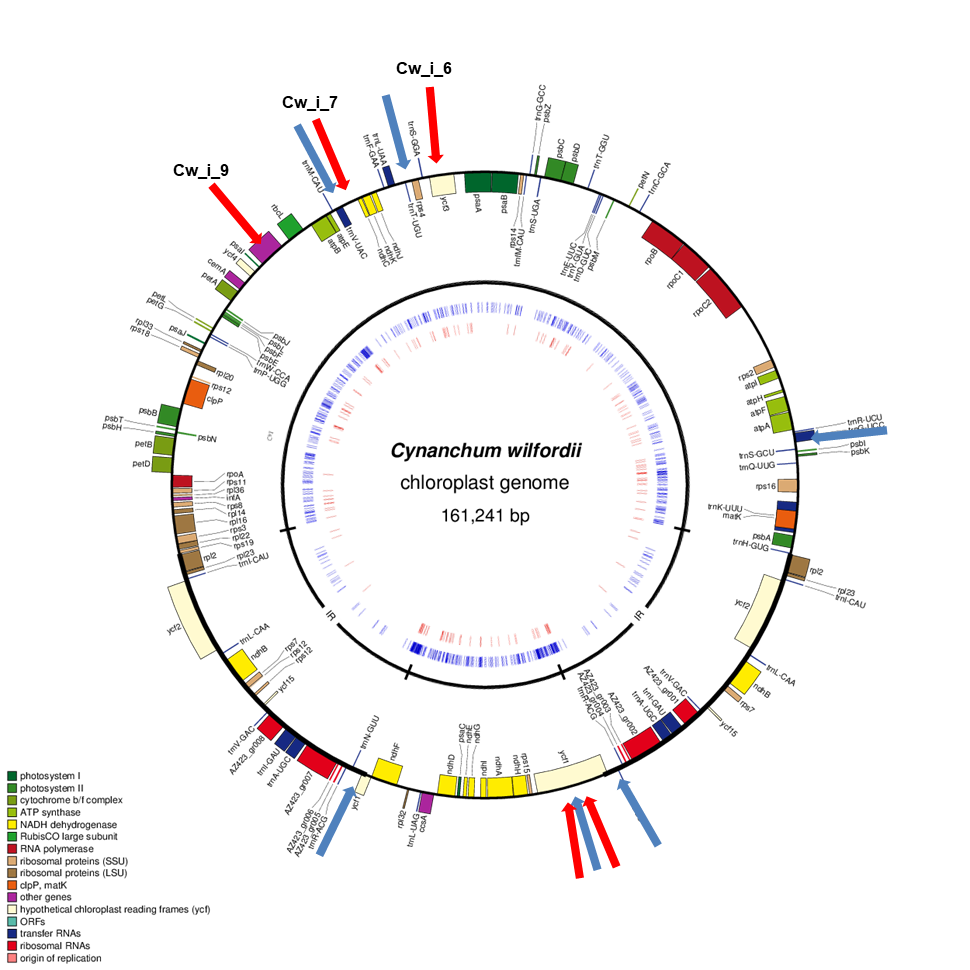
**

**Supplementary Fig. 9. Mitochondrial genome sizes and number of MTPTs in the 81 plants investigated in this study.** (a) Mitochondrial genome size distribution. (b) Distribution of MTPT lengths within the mitochondrial genomes. (c) Correlation plot for total MTPTs length and mitochondrial length of each species.


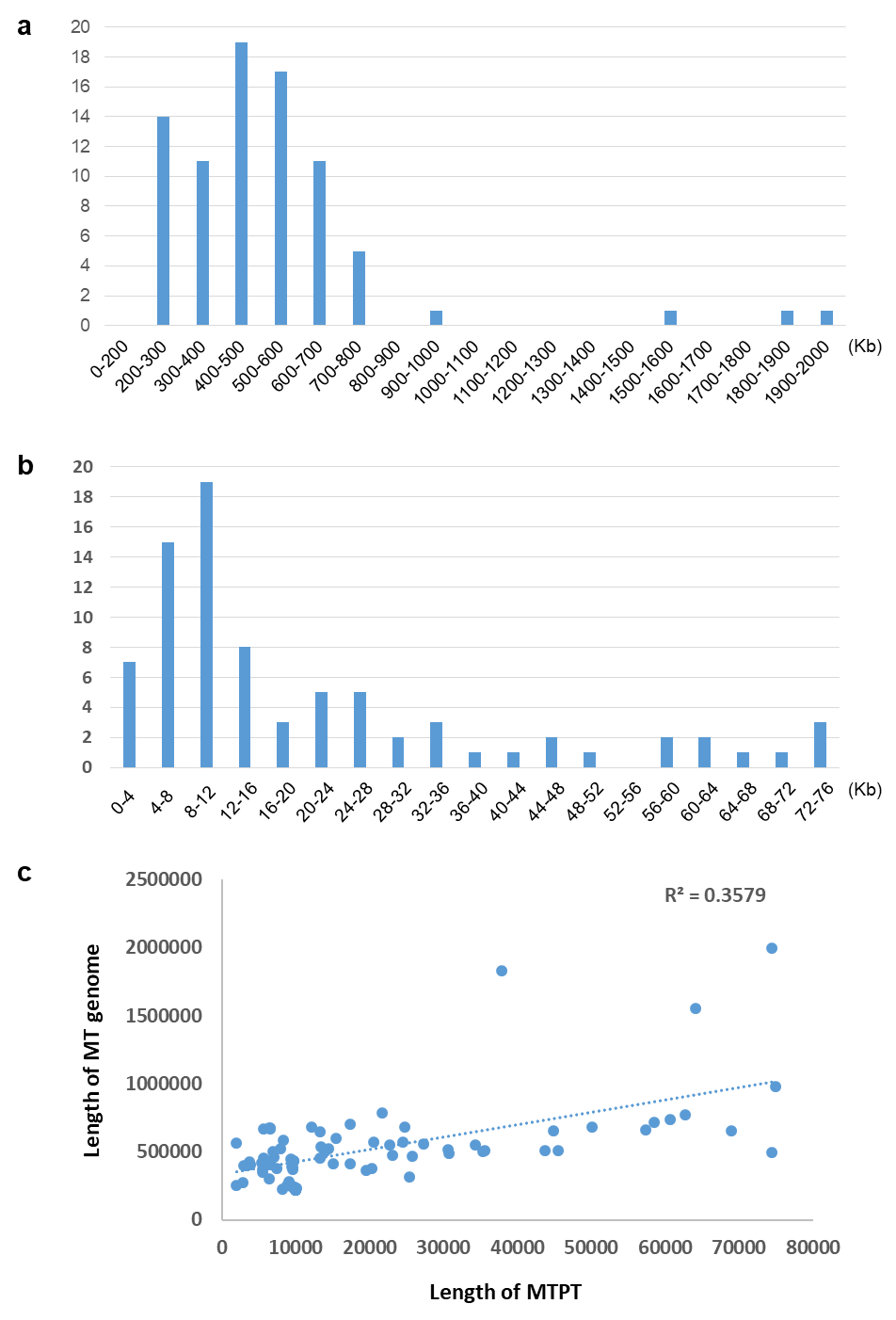


**Supplementary Figure S10. Full gel image for Figure 3. (a-c) Fig. 3b (c) Fig. 3c**


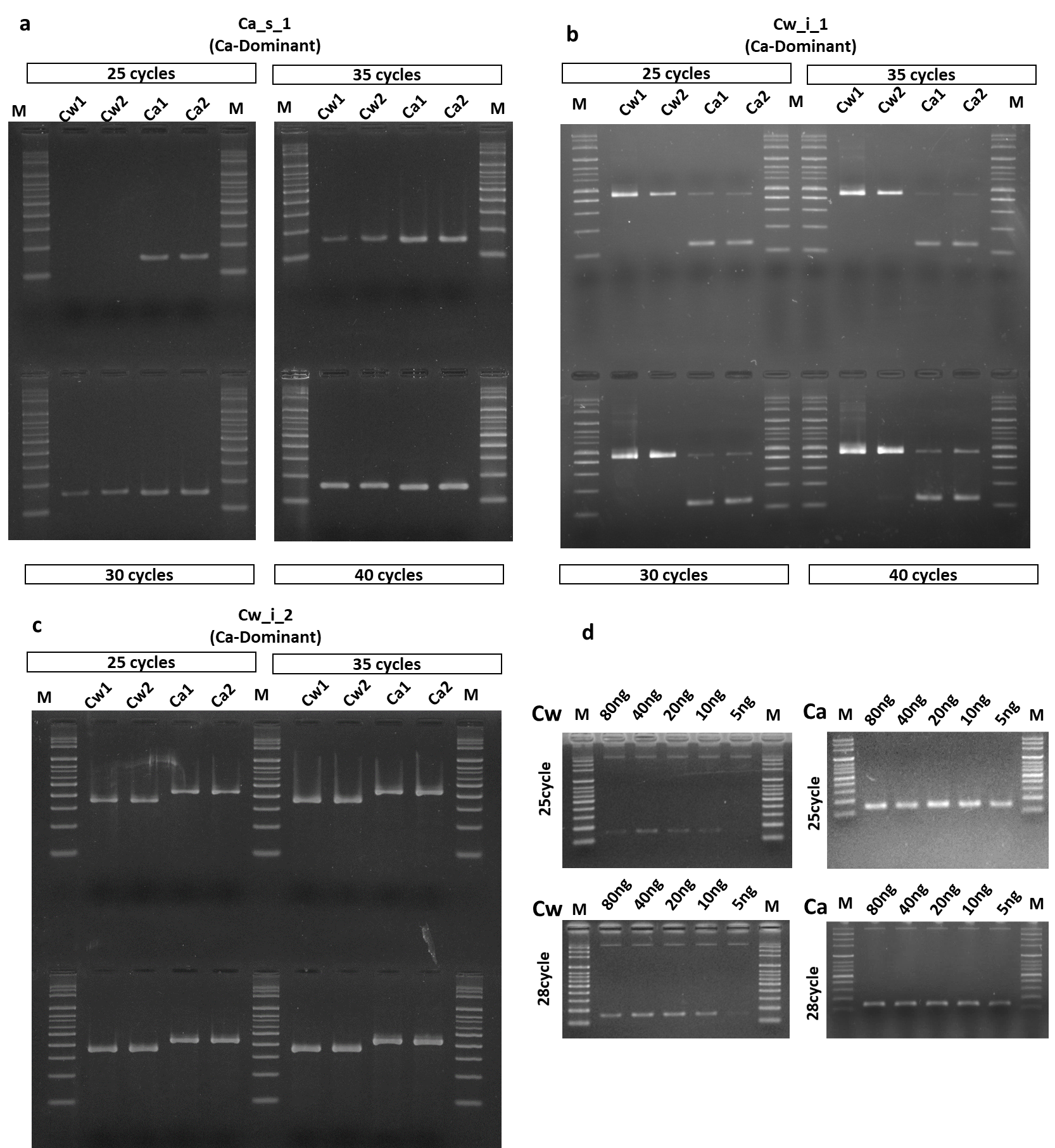


Supplementary Table 1. Plastid genes commonly transferred to the mitochondrial genome in *Cynanchum* species.

| **Gene product**  **(transferred genes/total genes)** | **Gene names** |
| --- | --- |
| ATP synthase (2/6) | *atpF*, *atpH* |
| Other proteins (3/6) | *matK**, *cemA*, *infA* |
| NADH oxidoreductase (9/11) | *ndhK*, *ndhE*, *ndhG*, *ndhI*,  *ndhA*, *ndhJ*, *ndhD*, |
| Cytochrome b6/f (5/6) | *petA*, *petD*, *petG*, *petL*, *petN*, |
| Photosystem I (4/7) | *psaA*, *psaB*, *psaC*, *psaI* |
| Photosystem II (7/16) | *psbA*, *psbC*, *psbD* |
| Large subunit ribosomal proteins (4/9) | *rpl22*, *rpl2*, *rpl36* |
| RNAP (3/4) | *rpoA*, *rpoB**, *rpoC2* |
| Small subunit ribosomal proteins (5/11) | *rps3*, *rps11*, *rps14*, *rps16* |
| Proteins of unknown function (3/3) | *ycf2*, *ycf4*, *ycf15* |

* Universal plant barcoding target regions in plastids

Supplementary Table 2. Sequence similarity of *matK* genes between species and between organelle genomes of *C. wilfordii* and *C. auriculatum.*

| **Similarity (%)**  **InDels/SNPs** | **Cw-Pt** | **Ca-Pt** | **Cw-MTPT** | **Ca-MTPT** |
| --- | --- | --- | --- | --- |
| Cw-Pt |  | 99.2 | 94.5 | 94.5 |
| Ca-Pt | 1/12 |  | 94.4 | 94.4 |
| Cw-MTPT | 11/79 | 12/82 |  | 99.6 |
| Ca-MTPT | 11/82 | 12/83 | 2/9 |  |

Cw, *C. wilfordii*; Ca, *C. auriculatum*;Pt, plastid genome; MTPT, mitochondrial DNA of plastid origin

Numbers of SNPs/InDels and nucleotide similarity (%) are shown below and above the self-comparison diagonal, respectively

Supplementary Table 3. Mode values of synonymous substitution (Ks) among four Apocynaceae species calculated based on plastid and mitochondrial genes.

| **Pt**  **Mt** | **Cw** | **Ca** | **As** | **Rs** |
| --- | --- | --- | --- | --- |
| Cw |  | 0.006 - 0.012 | 0.03-0.06 | 0.09-0.12 |
| Ca | 0 |  | 0.03-0.06 | 0.09-0.12 |
| As | 0.012-0.018 | 0.012-0.018 |  | 0.09-0.12 |
| Rs | 0.021-0.035 | 0.021-0.035 | 0.021-0.035 |  |

Cw, *C. wilfordii*; Ca, *C. auriculatum*; As, *Asclepias syriaca*; Rs, *Rhazya stricta*; Pt, Plastid genome; MTPT, Mitochondrial DNA of plastid origin

Mode values of Ks between mitochondrial genes and between plastid genes are shown below and above the self-comparison diagonal, respectively. Ks values between MTPT genes were calculated only between Cw and Ca, with an average value of 0.0001

Supplementary Table 4. List of the plant mitochondrial genomes used in this study.

| **#** | **Name** | **NCBI accession** | **#** | **Name** | **NCBI accession** |
| --- | --- | --- | --- | --- | --- |
| 1 | *Butomus umbellatus* | KC208619.1 | 46 | *Cannabis sativa* | KU310670.1 |
| 2 | *Spirodela polyrhiza* | NC_017840.1 | 47 | *Glycine max* | JX463295.1 |
| 3 | *Phoenix dactylifera* | JN375330.1 | 48 | *Vigna radiata* | HM367685.1 |
| 4 | *Cocos nucifera* | KX028885.1 | 49 | *Millettia pinnata* | JN872550.1 |
| 5 | *Allium cepa* | KU318712.1 | 50 | *Lotus japonicus* | NC_016743.2 |
| 6 | *Oryza minuta* | KU176938.1 | 51 | *Medicago truncatula* | KT971339.1 |
| 7 | *Oryza rufipogon* | NC_013816.1 | 52 | *Vicia faba* | KC189947.1 |
| 8 | *Oryza sativa* subsp. Japonica | NC_011033.1 | 53 | *Vitis vinifera* | NC_012119.1 |
| 9 | *Bambusa oldhamii* | EU365401.1 | 54 | *Geranium maderense* | KP940515.1 |
| 10 | *Sorghum bicolor* | DQ984518.1 | 55 | *Beta macrocarpa* | FQ378026.1 |
| 11 | *Zea luxurians* | DQ645537.1 | 56 | *Beta vulgaris* subsp. maritima | FP885834.1 |
| 12 | *Zea mays* subsp. mays | NC_007982.1 | 57 | *Silene latifolia* | HM562727.1 |
| 13 | *Zea perennis* | DQ645538.1 | 58 | *Silene vulgaris* | JF750427.1 |
| 14 | *Tripsacum dactyloides* | NC_008362.1 |  |  | JF750428.1 |
| 15 | *Lolium perenne* | JX999996.1 |  |  | JF750429.1 |
| 16 | *Triticum aestivum* | AP008982.1 |  |  | JF750430.1 |
| 17 | *Brassica carinata* | JF920287.1 | 59 | *Daucus carota* subsp. sativus | JQ248574.1 |
| 18 | *Brassica nigra* | KP030753.1 | 60 | *Helianthus annuus* | KF815390.1 |
| 19 | *Sinapis arvensis* | KM851044.1 | 61 | *Vaccinium macrocarpon* | KF386162.1 |
| 20 | *Eruca vesicaria* subsp. sativa | KF442616.1 | 62 | *Viscum album* | KJ129610.1 |
| 21 | *Raphanus sativus* | JQ083668.1 | 63 | *Rhazya stricta* | KJ485850.1 |
| 22 | *Brassica juncea* | NC_016123.1 | 64 | *Asclepias syriaca* | KF541337.1 |
| 23 | *Brassica rapa* subsp. campestris | JF920285.1 | 65 | *Cynanchum wilfordii* | MH931257 |
| 24 | *Brassica oleracea* | KJ820683.1 |  |  | MH931258 |
| 25 | *Brassica napus* | NC_008285.1 |  |  | MH931259 |
| 26 | *Schrenkiella parvula* | KT988071.2 | 66 | *Cynanchum auriculatum* | MH931260 |
| 27 | *Arabidopsis thaliana* | NC_037304.1 | 67 | *Hesperelaea palmeri* | KX545367.1 |
| 28 | *Batis maritima* | KJ820684.1 | 68 | *Boea hygrometrica* | JN107812.1 |
| 29 | *Carica papaya* | EU431224.1 | 69 | *Mimulus guttatus* | JN098455.1 |
| 30 | *Corchorus capsularis* | KT894204.1 | 70 | *Castilleja paramensis* | KT959112.1 |
| 31 | *Corchorus olitorius* | KT894205.1 | 71 | *Salvia miltiorrhiza* | KF177345.1 |
| 32 | *Gossypium barbadense* | KP898249.1 | 72 | *Ajuga reptans* | KF709392.1 |
| 33 | *Gossypium harknessii* | JX944506.1 | 73 | *Capsicum annuum* | KJ865409.1 |
| 34 | *Gossypium hirsutum* | JX944505.1 | 74 | *Solanum lycopersicum* | NC_035963.1 |
| 35 | *Gossypium raimondii* | KU317325.1 | 75 | *Solanum tuberosum* | MF989953.1 |
| 36 | *Ricinus communis* | HQ874649.1 |  |  | MF989954.1 |
| 37 | *Populus tremula* | KT337313.1 |  |  | MF989955.1 |
| 38 | *Salix purpurea* | KU198635.1 |  |  | MF989956.1 |
| 39 | *Salix suchowensis* | KU056812.1 |  |  | MF989957.1 |
| 40 | *Citrullus lanatus* | GQ856147.1 | 76 | *Solanum commersonii* | MF989960.1 |
| 41 | *Cucumis sativus* | NC_016004.1 |  |  | MF989961.1 |
|  |  | NC_016005.1 | 77 | *Hyoscyamus niger* | KM207685.1 |
|  |  | NC_016006.1 | 78 | *Nicotiana tabacum* | NC_006581.1 |
| 42 | *Cucurbita pepo* | GQ856148.1 | 79 | *Nicotiana sylvestris* | KT997964.1 |
| 43 | *Malus hupehensis* | KR534606.1 | 80 | *Liriodendron tulipifera* | KC821969.1 |
| 44 | *Malus x domestica* | NC_018554.1 | 81 | *Nelumbo nucifera* | KR610474.1 |

Supplementary Table 5. Number of polymorphic sites between the plastid genomes of *C. wilfordii* and *C. auriculatum*.

| InDel  SNP | Cw1 | Cw2 | Cw3 | Cw4 | Cw5 | Ca |
| --- | --- | --- | --- | --- | --- | --- |
| Cw1 |  | 7 | 6 | 7 | 7 | 253 |
| Cw2 | 3 |  | 1 | 2 | 4 | 255 |
| Cw3 | 5 | 2 |  | 1 | 3 | 254 |
| Cw4 | 4 | 1 | 3 |  | 4 | 253 |
| Cw5 | 3 | 0 | 2 | 1 |  | 254 |
| Ca | 971 | 968 | 971 | 970 | 968 |  |

Cw, *C. wilfordii*; Cw1, NC_029459.1; Cw2, MK182385 Cw3, MK182386; Cw4, MK182387; Cw5 MK182388.

Ca, *C. auriculatum,* NC_029460.1.

The number of SNP and InDel sites is shown below and above the self-comparison diagonal, respectively.

Supplementary Table 6. Plastid DNA markers used for authentication of *Cynanchum* species.

| **Primer** | **Sequence (5ʹ to 3ʹ)** | **Gene name** | **Product size (bp)** | |
| --- | --- | --- | --- | --- |
|  |  |  | ***C. wilfordii*** | ***C. auriculatum*** |
| Cw_i_1 | F: TCGGCAAGAACAATTCCTGT  R: TGACCGTCCAATTTGTGTAGA | *rps2* – *rpoC2* | 481 | 135 |
| Cw_i_2 | F: AGATGATCTAGCAACGATGGGA  R: CGGGTATTCAAGCGGATTGG | *rpoB*­­ – *trnC-GCA* | 347 | 428 |
| Cw_i_3 | F: TACACAAGCACGACAGGTCC  R: CGGTTCGAGTCCGTATAGCC | *ndhC* – *trnV*-UAC | 468 | 407 |
| Cw_i_4 | F: ACTCGGCCCAATCTTTTCCT  R: TGTGGATTCAAGACAACAAT | *rbcL* – *accD* | 230 | 310 |
| Cw_i_5 | F: GTCTGAGACGGCCCAGAAAG  R: CCCGAAAGAACCGGACATGA | *petD* intron | 269 | 301 |
| Cw_i_6 | F: CCTTGGTGCCGCGTTTTAAT  R: CGTTATTCTTCTGACGGTGGGA | *ycf3 intron* | 207 | 181 |
| Cw_i_7 | F: TGATCGAATTGACTAGTTTCCTTTG  R: GGACCTGTCGTGCTTGTGTA | *ndhC - trnV-UAC* | 839 | 819 |
| Cw_i_8 | F: TAATGGCGAAACGGAGGGTT  R: GAATTAACTCACTATCTTGCATC | *accD* | 311 | 347 |
| Ca_s_1 | F: AATGAGAAAAGTTTCTG  R: CTTGTTCCAATTATTCC | *matK* | 151 | 151 |
| Cw_s_2 | F: GCCGAATCCTTCTAGAGCCC  R: CGGACGTTCCAGTGGACATT | *rpoB* | 115 | 115 |
| Cw_s_3 | F: AGCGATCTTTTCGTAGACGTT  R: TTCCCTTGTTCACTAATAAATCGAC | *rps18* | 100 | 100 |
| Cw_s_4 | F: CCAAGACGAACTAATGCAGGG  R: TTGCGACACCCATCAAAGGA | *psbH* | 100 | 100 |
| Cw_s_5 | F: CATTCCCGCAGGAGATCCG  R: ACTCCAGGGATGAATCGAAAAAGA | *ycf2* | 120 | 120 |
| Cw_s_6 | F: AACCAATAGCGATTCATACAAGC  R: TGGATTTGGATAAAGAGAACCATCT | *ycf1* | 118 | 118 |
| Cw_s_7 | F: GCAAATTGATAAAACCCGGCG  R: AGAGTTGAAGCCCCAAAGGG | *ndhH* | 103 | 103 |
